# Supplementary figures and images for: Comparison of the synergistic effect of lipid nanobubbles and SonoVue microbubbles for high intensity focused ultrasound thermal ablation of tumors
Source: PeerJ. 2016 Feb 22;4:e1716. doi: 10.7717/peerj.1716 (PMC4768712; doi:10.7717/peerj.1716)

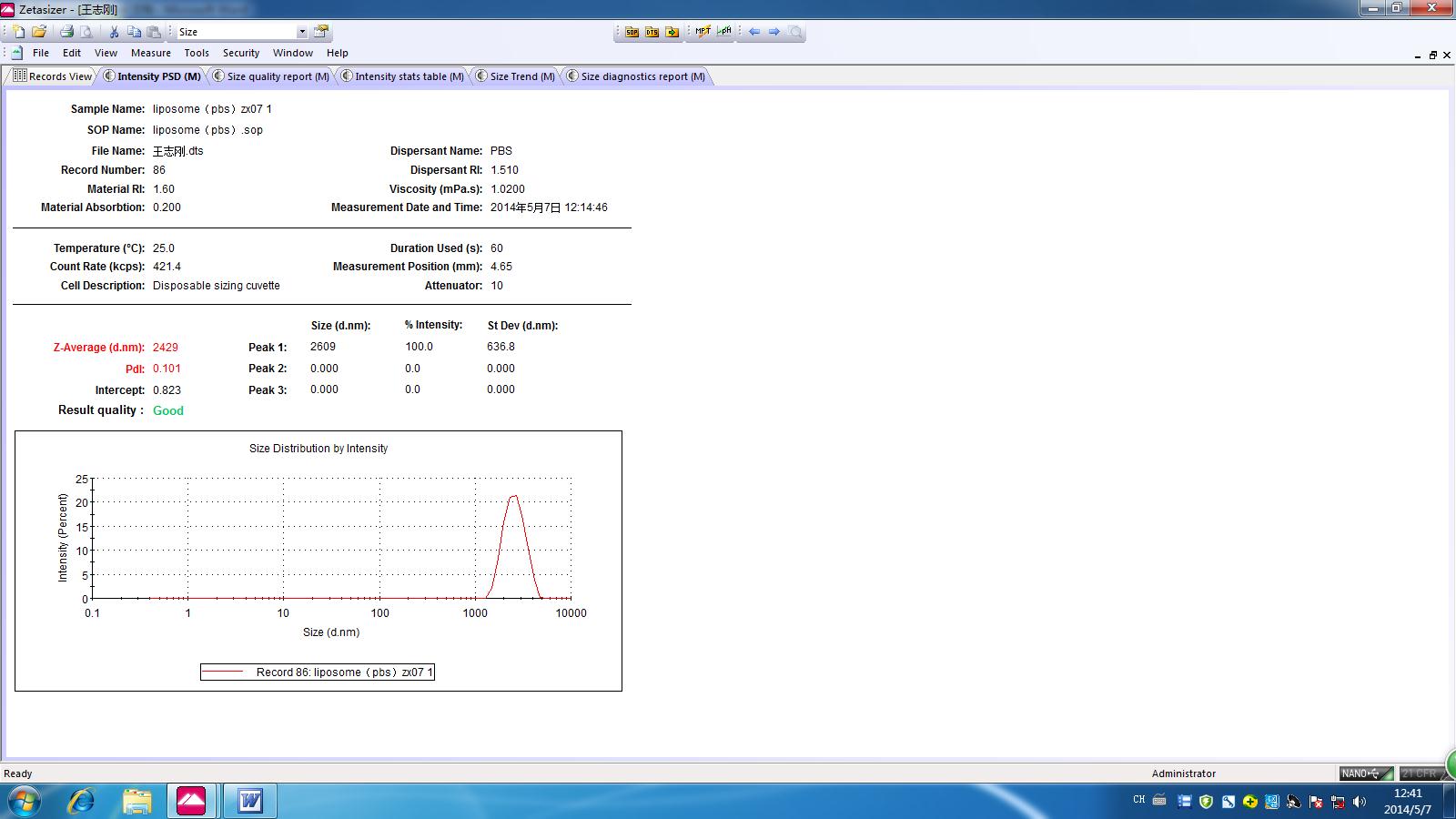

Supplement: Data S1 [file peerj-04-1716-s001.zip › characterization of NBs and MBs/MBs size.jpg]

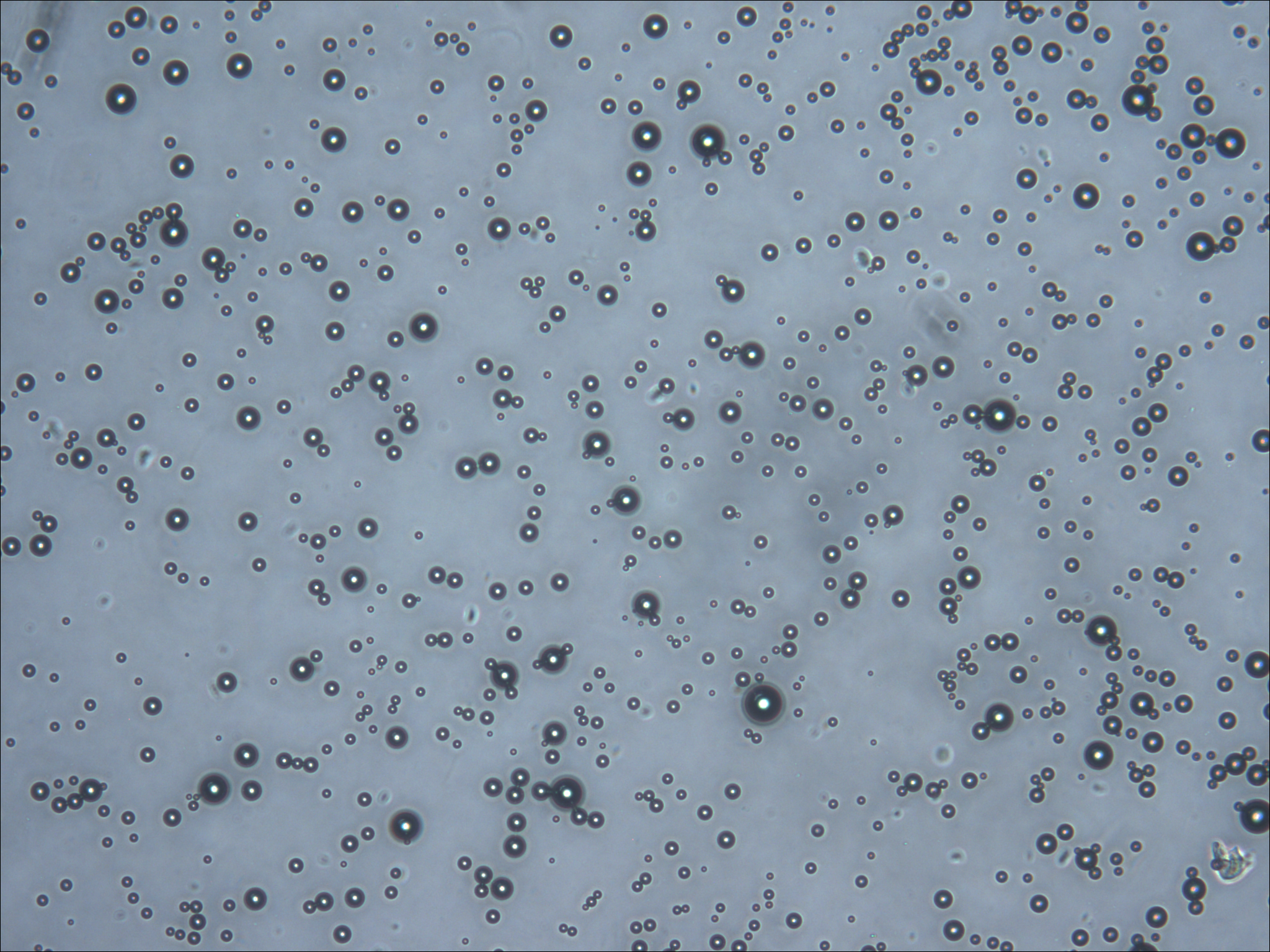

Supplement: Data S1 [file peerj-04-1716-s001.zip › characterization of NBs and MBs/MBs.png]

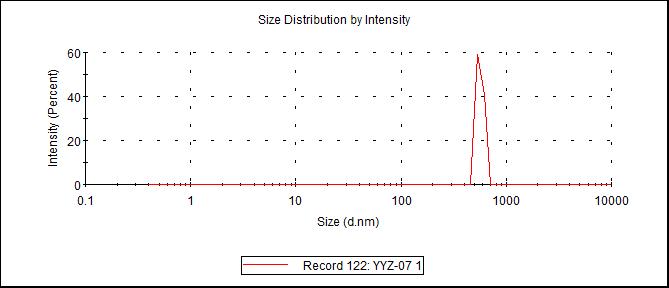

Supplement: Data S1 [file peerj-04-1716-s001.zip › characterization of NBs and MBs/NBs size.jpg]

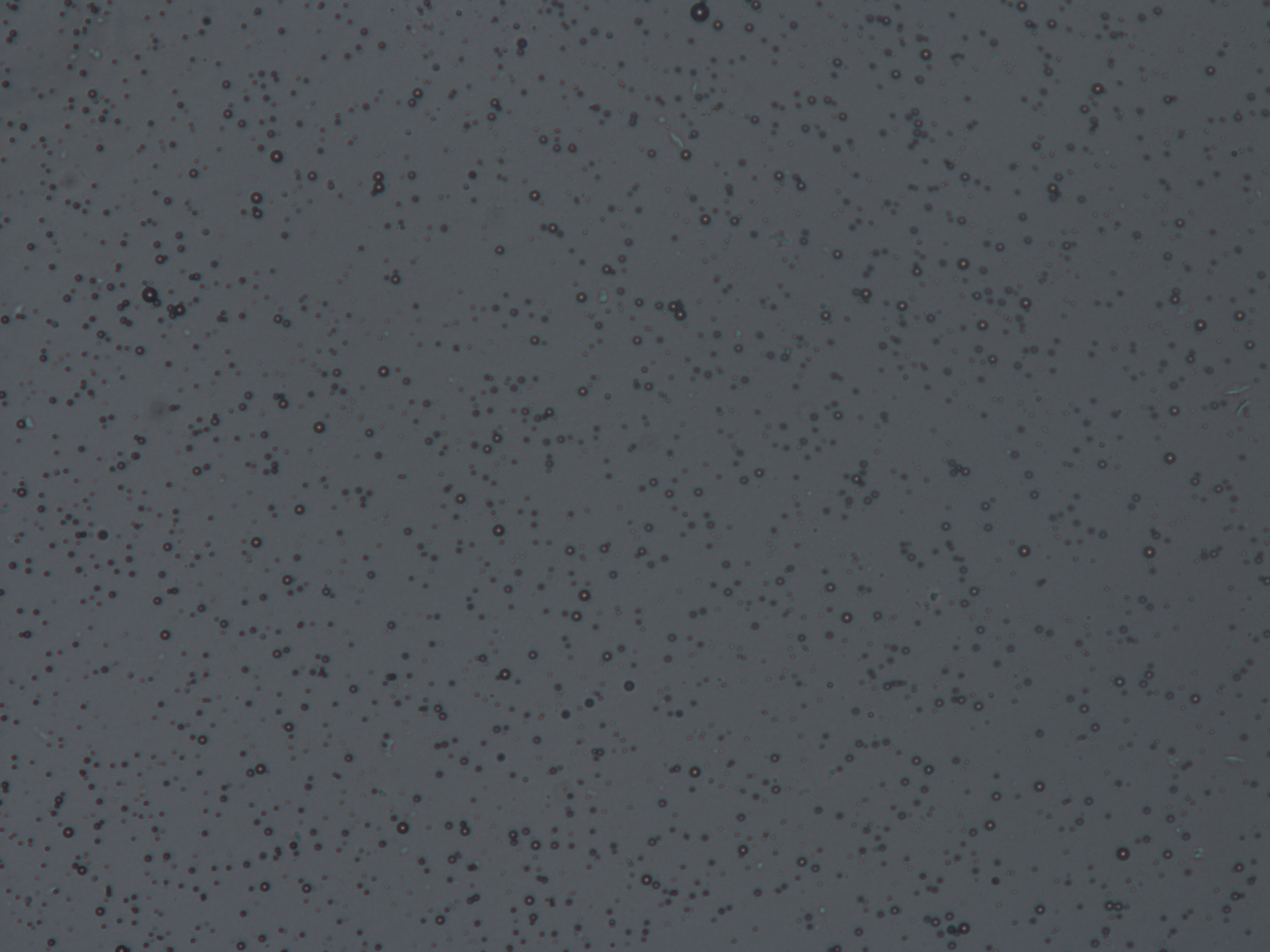

Supplement: Data S1 [file peerj-04-1716-s001.zip › characterization of NBs and MBs/NBs.png]

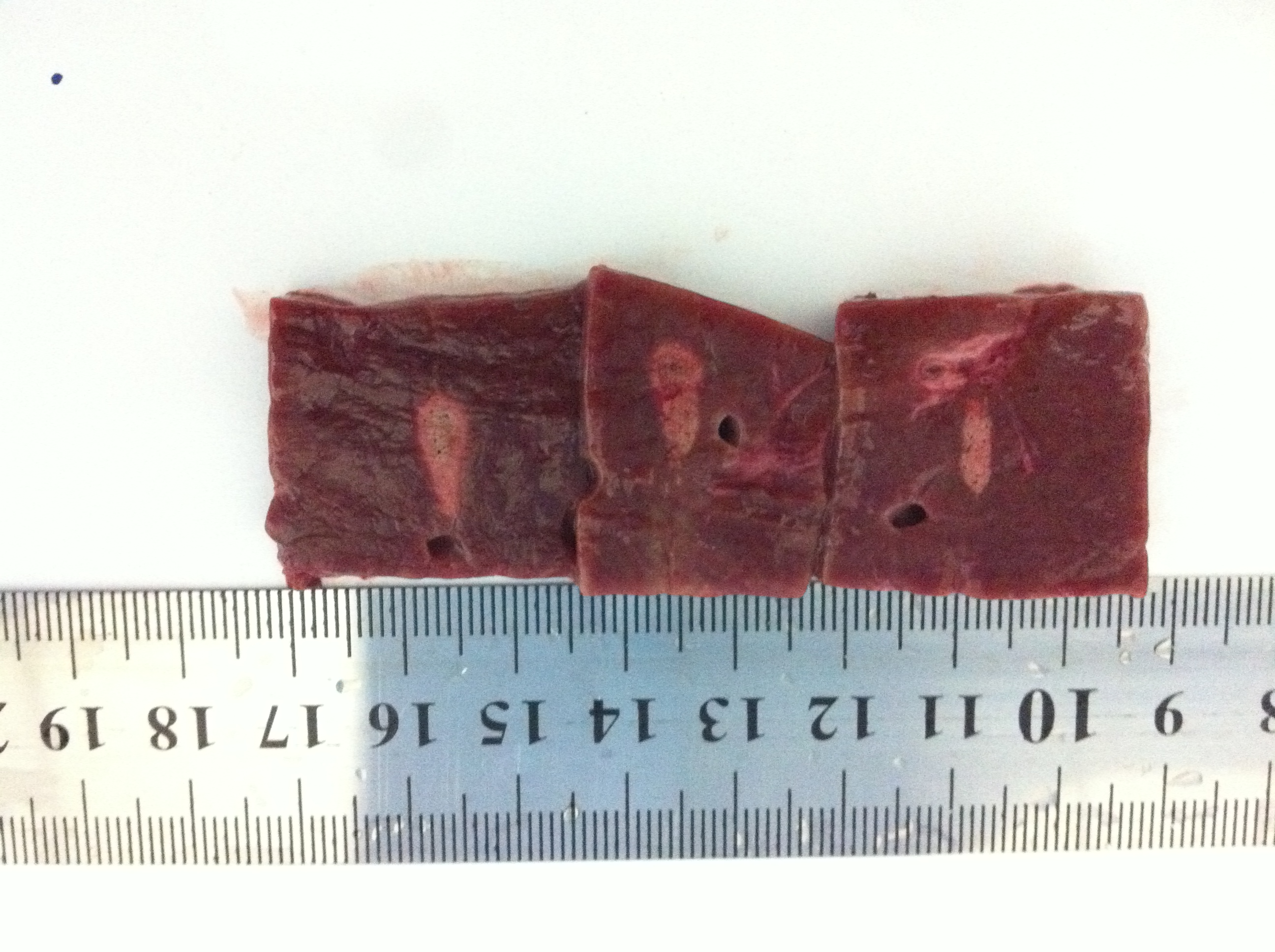

Supplement: Data S2 [file peerj-04-1716-s002.zip › raw data of excised bovine liver/IMG_1545.JPG]

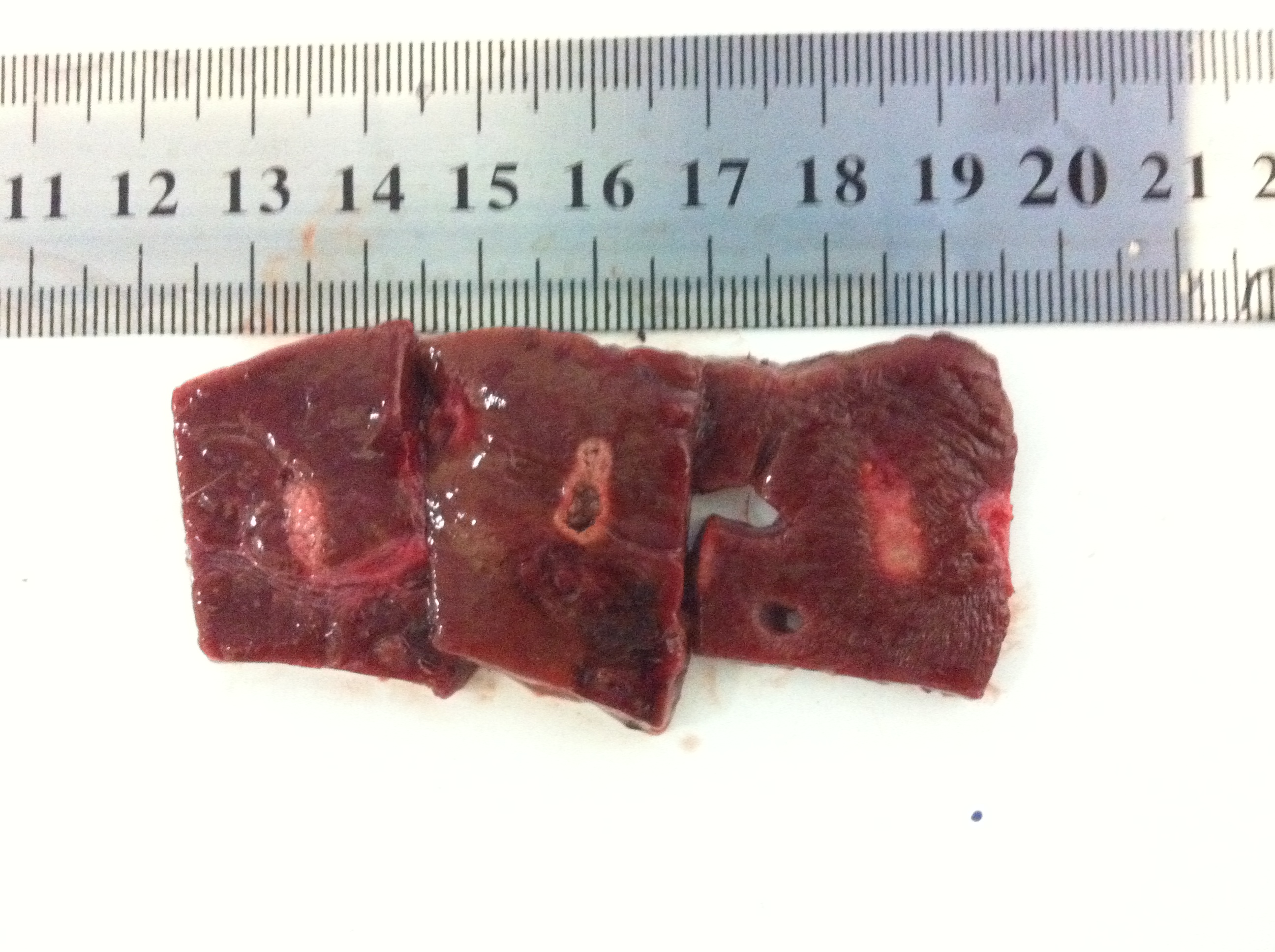

Supplement: Data S2 [file peerj-04-1716-s002.zip › raw data of excised bovine liver/IMG_1554.JPG]

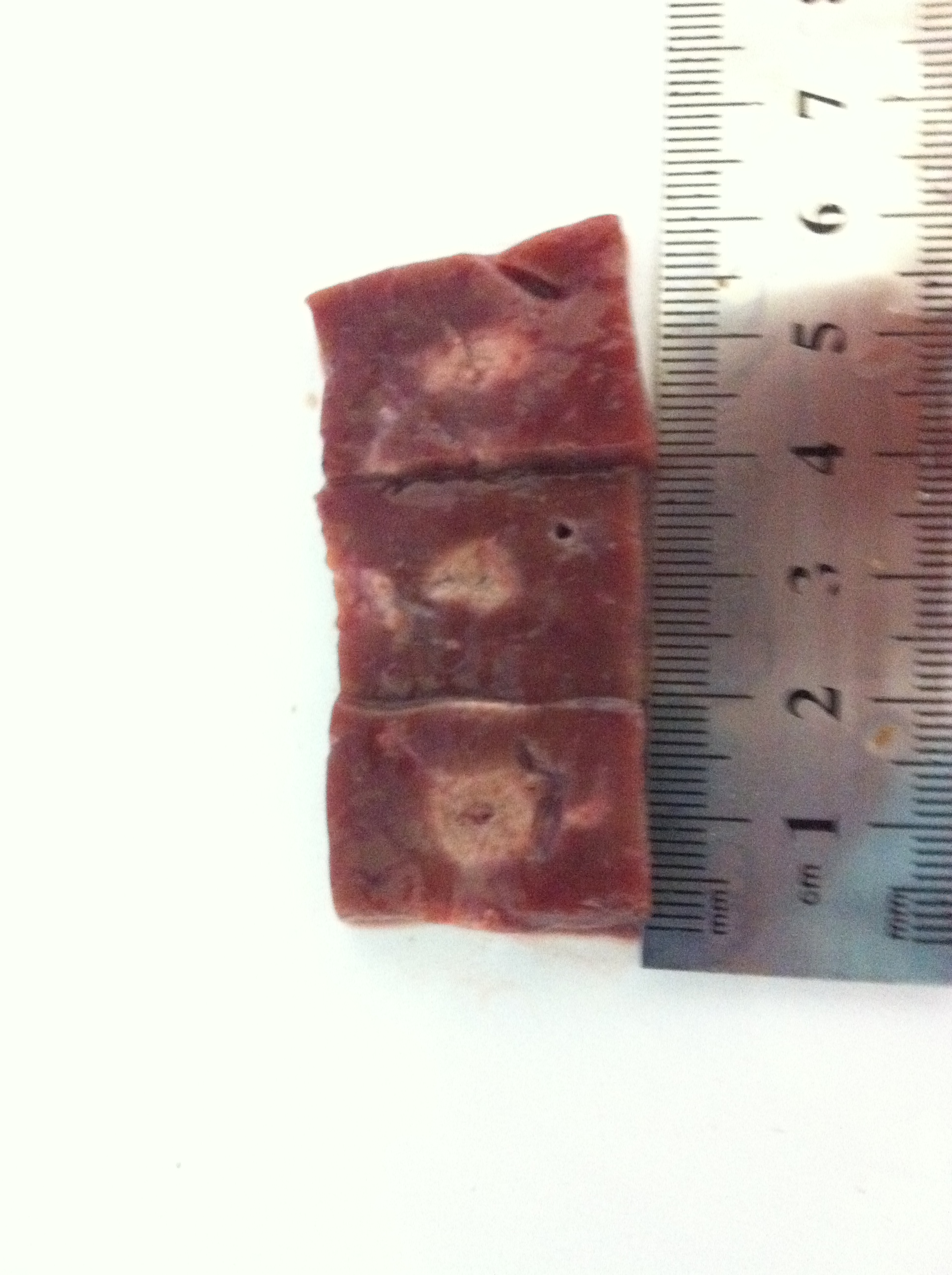

Supplement: Data S2 [file peerj-04-1716-s002.zip › raw data of excised bovine liver/IMG_1629.JPG]

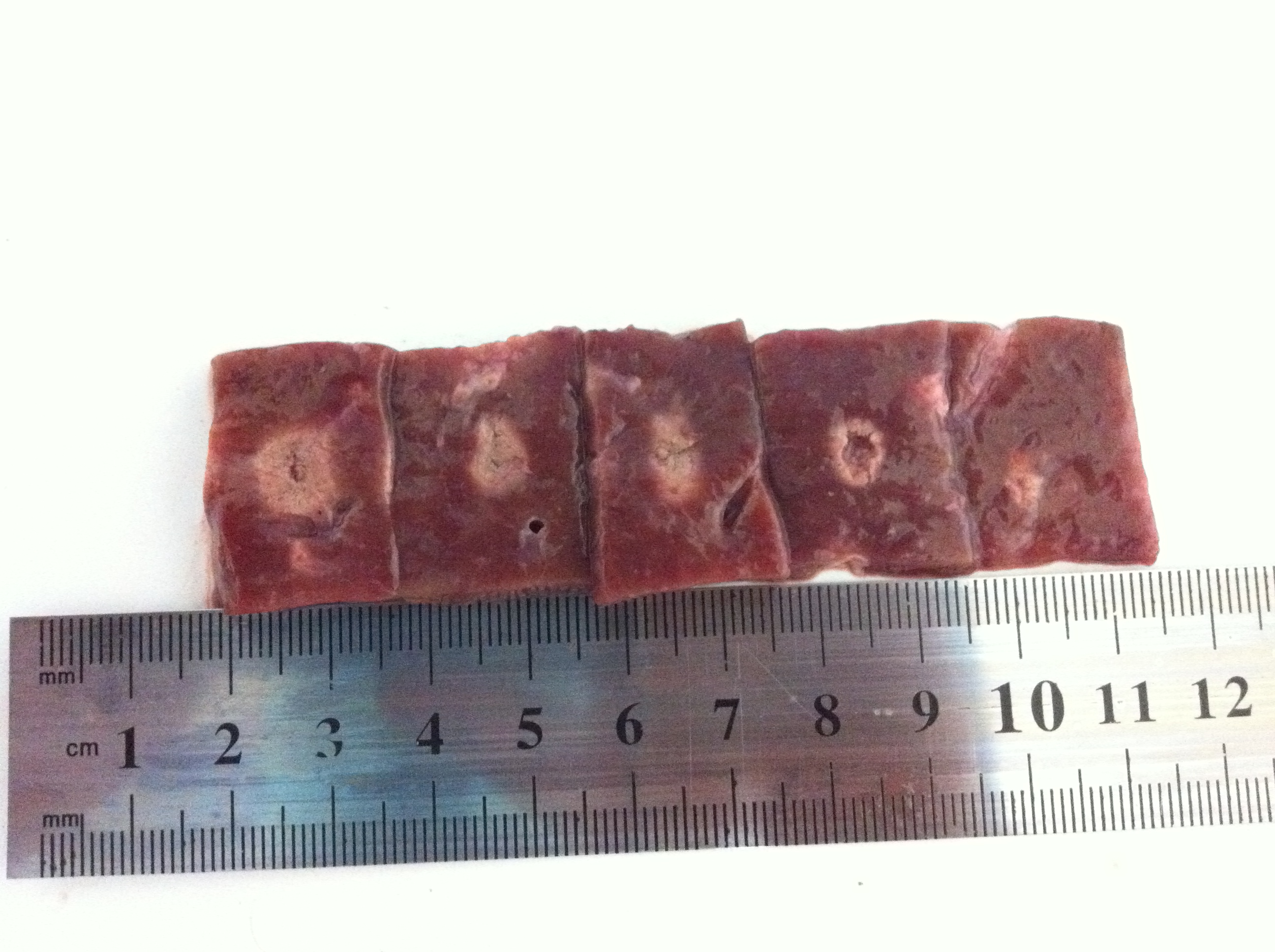

Supplement: Data S2 [file peerj-04-1716-s002.zip › raw data of excised bovine liver/IMG_1634.JPG]

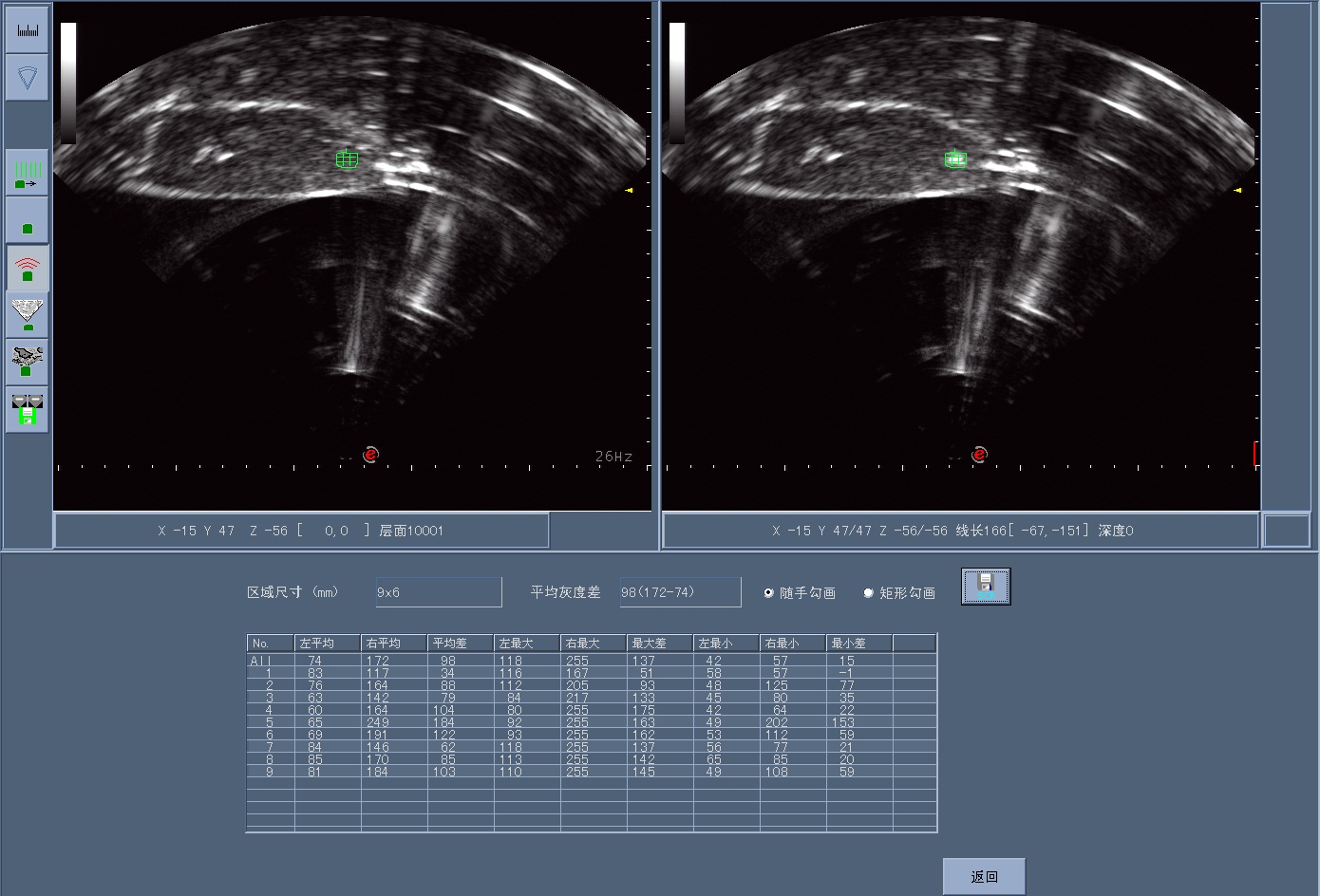

Supplement: Data S2 [file peerj-04-1716-s002.zip › raw data of excised bovine liver/mb.jpg]

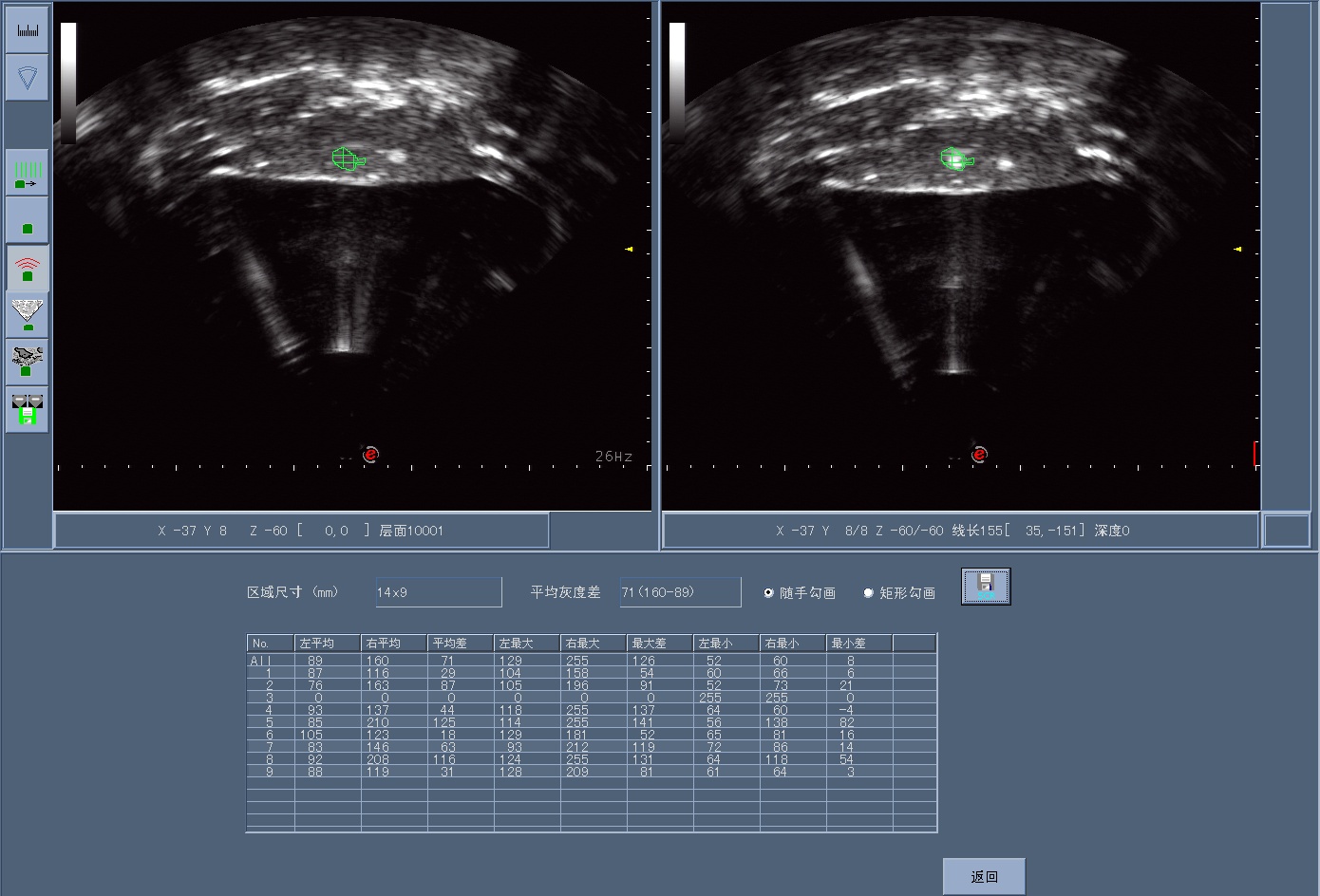

Supplement: Data S2 [file peerj-04-1716-s002.zip › raw data of excised bovine liver/MBs,150w,5s.jpg]

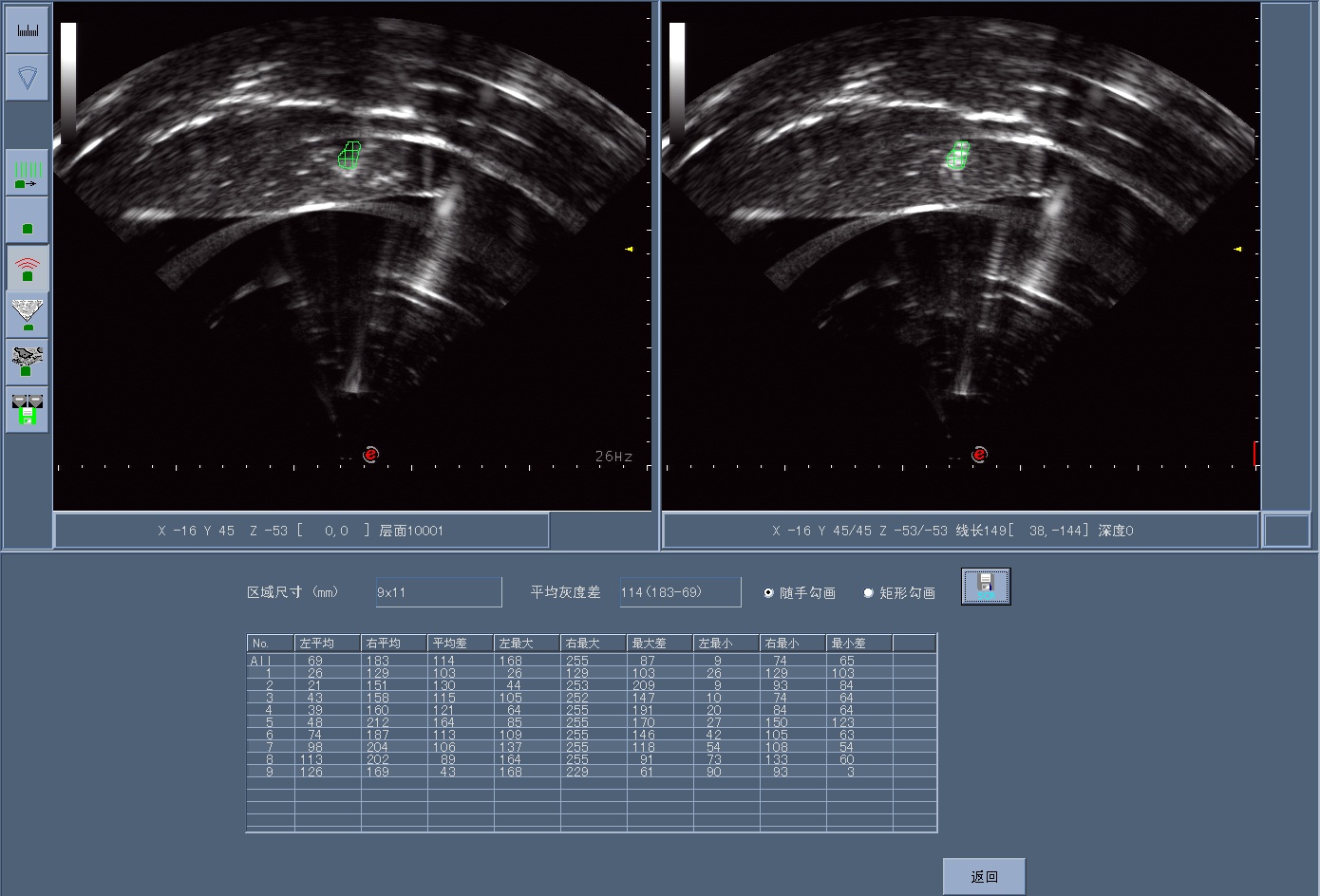

Supplement: Data S2 [file peerj-04-1716-s002.zip › raw data of excised bovine liver/MBs,180w,5s.jpg]

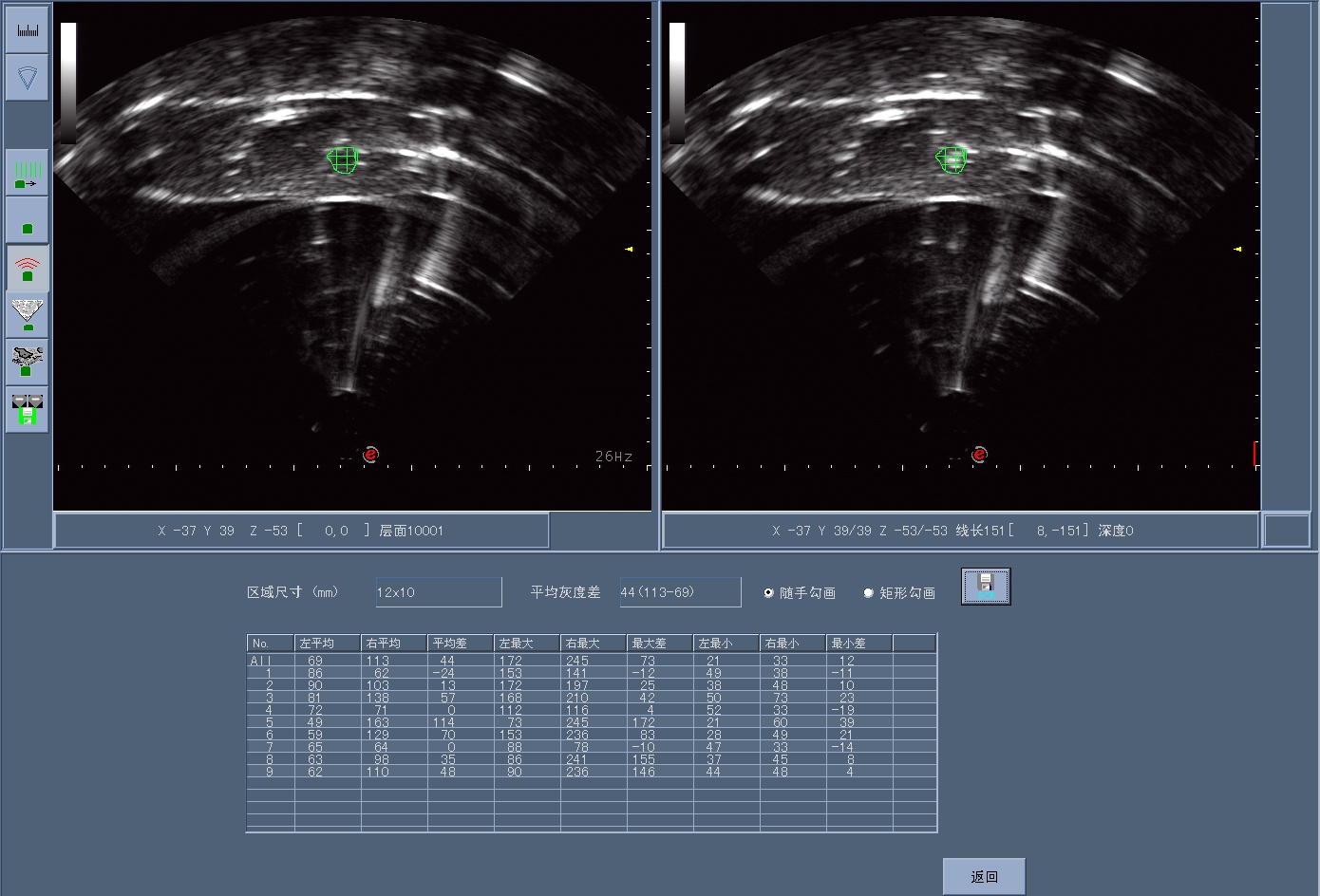

Supplement: Data S2 [file peerj-04-1716-s002.zip › raw data of excised bovine liver/NBs,120w,5s.jpg]

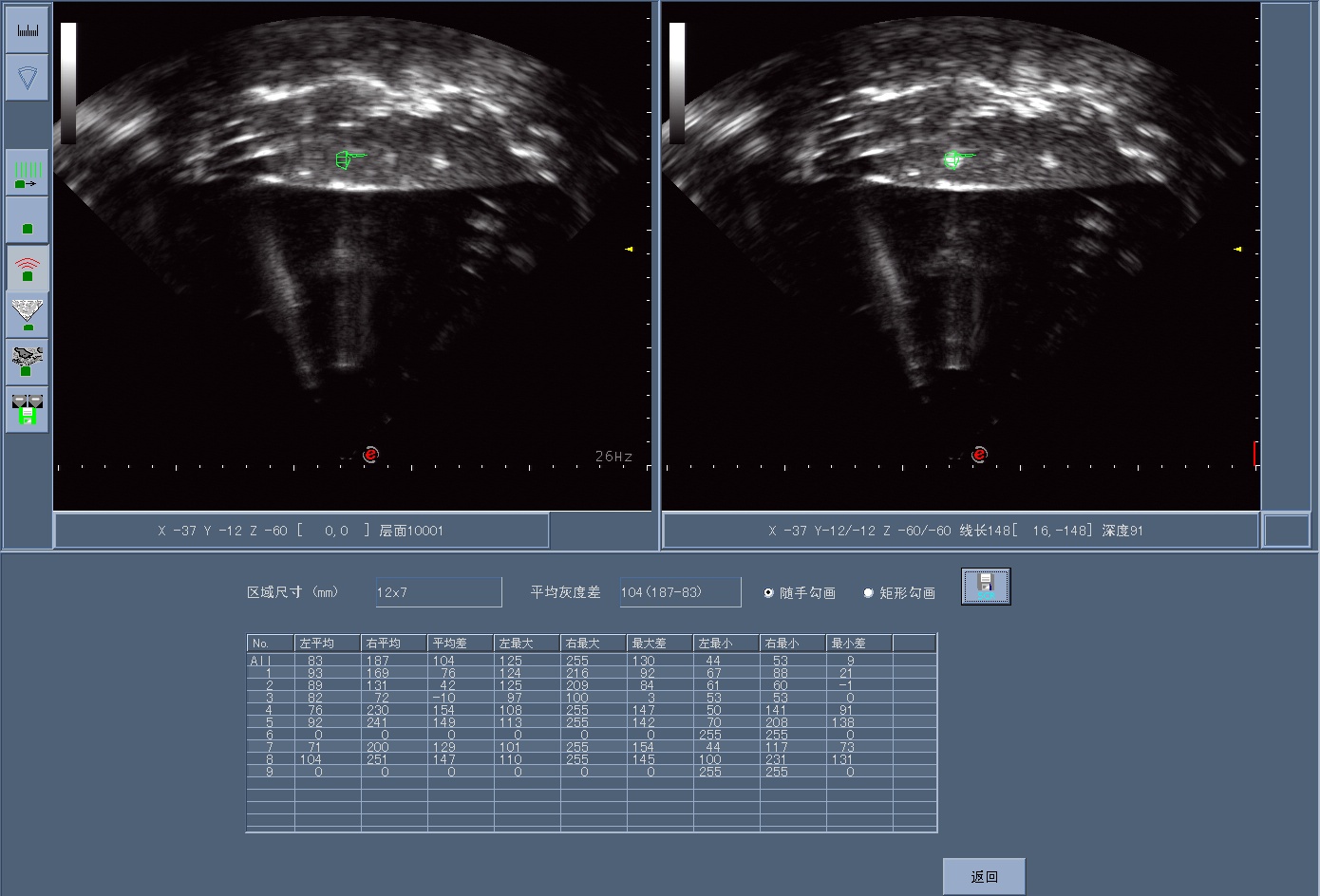

Supplement: Data S2 [file peerj-04-1716-s002.zip › raw data of excised bovine liver/NBs,150w,5s.jpg]

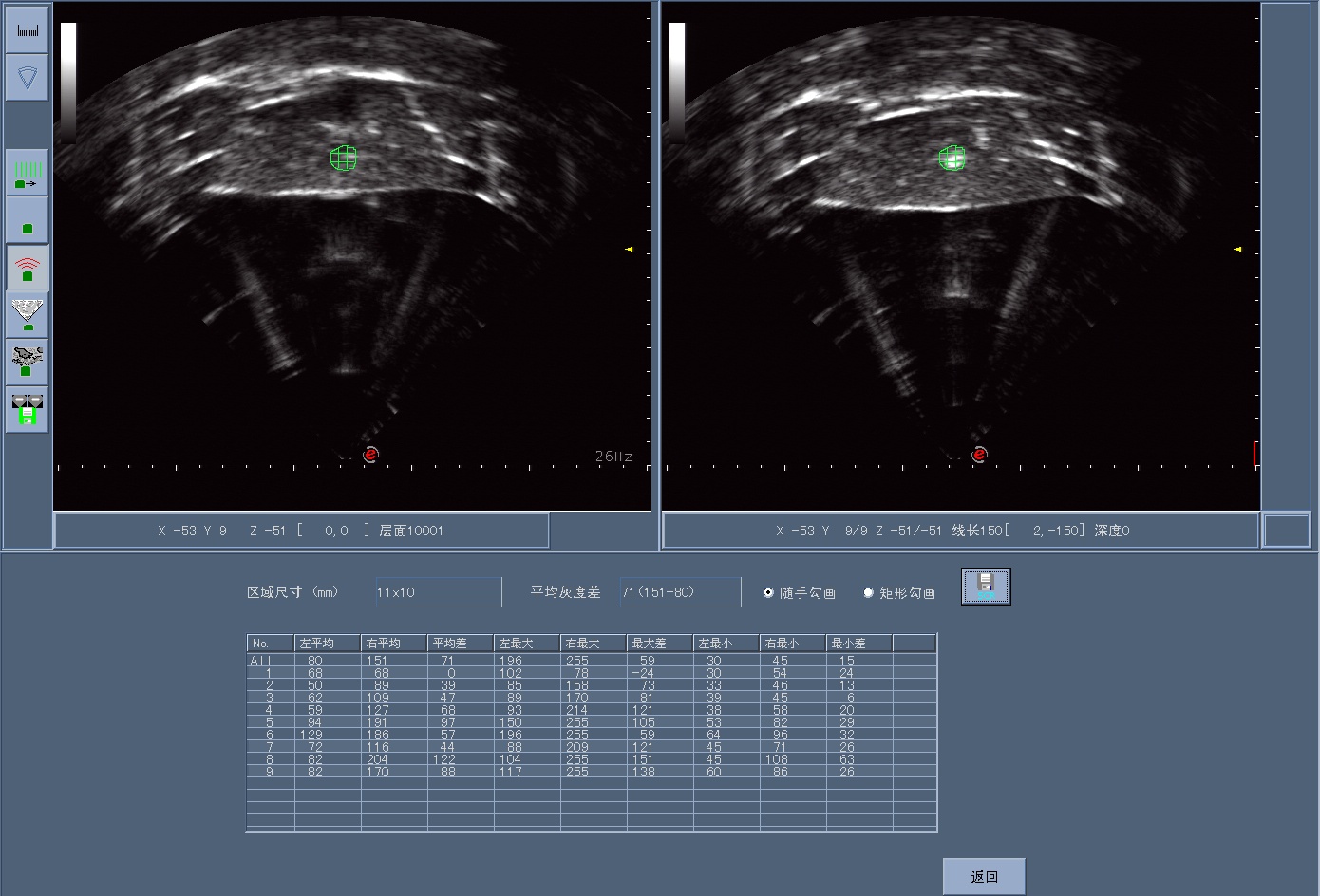

Supplement: Data S2 [file peerj-04-1716-s002.zip › raw data of excised bovine liver/NBs,180wú1⁄45s.jpg]

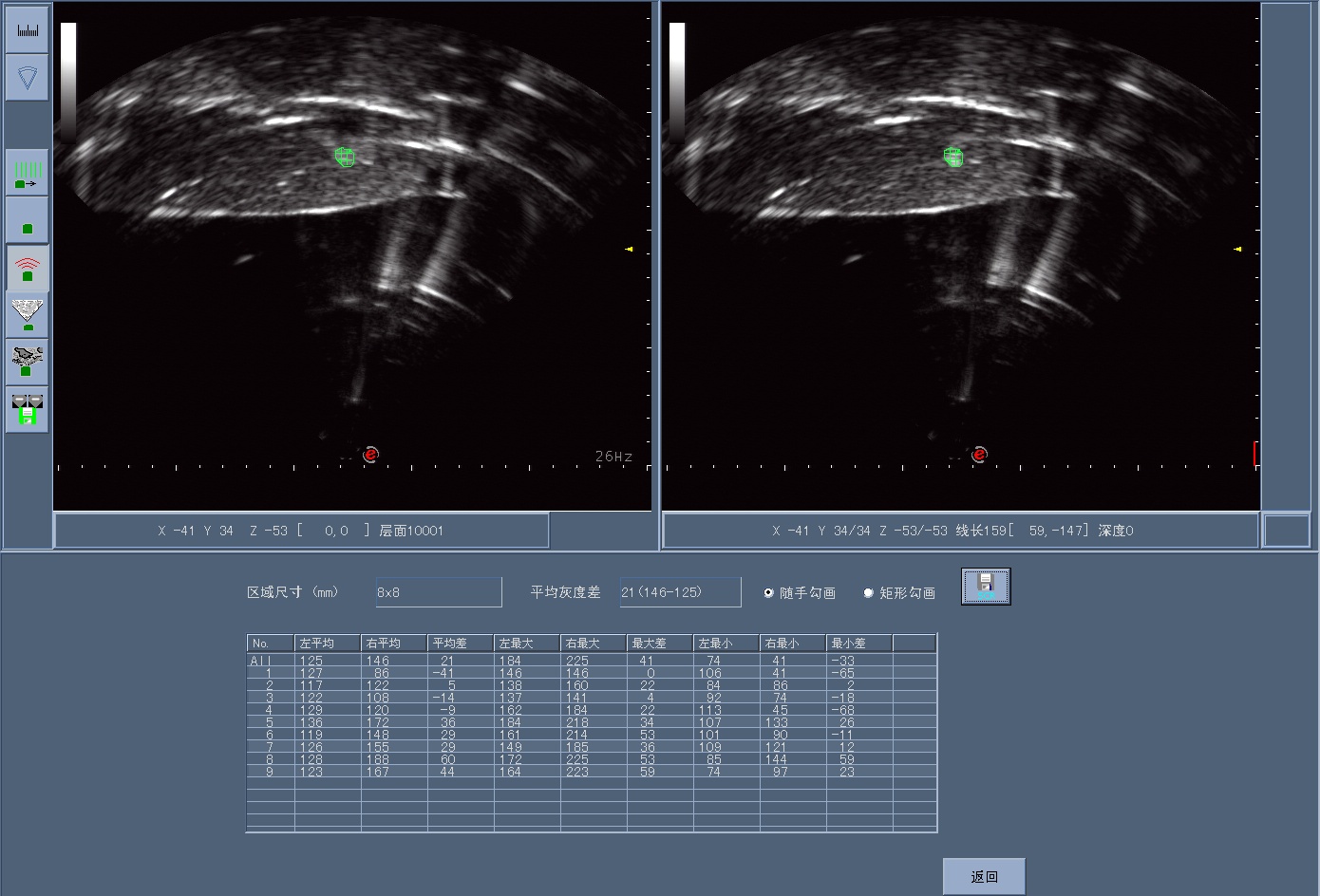

Supplement: Data S2 [file peerj-04-1716-s002.zip › raw data of excised bovine liver/PBS,120w,5s.jpg]

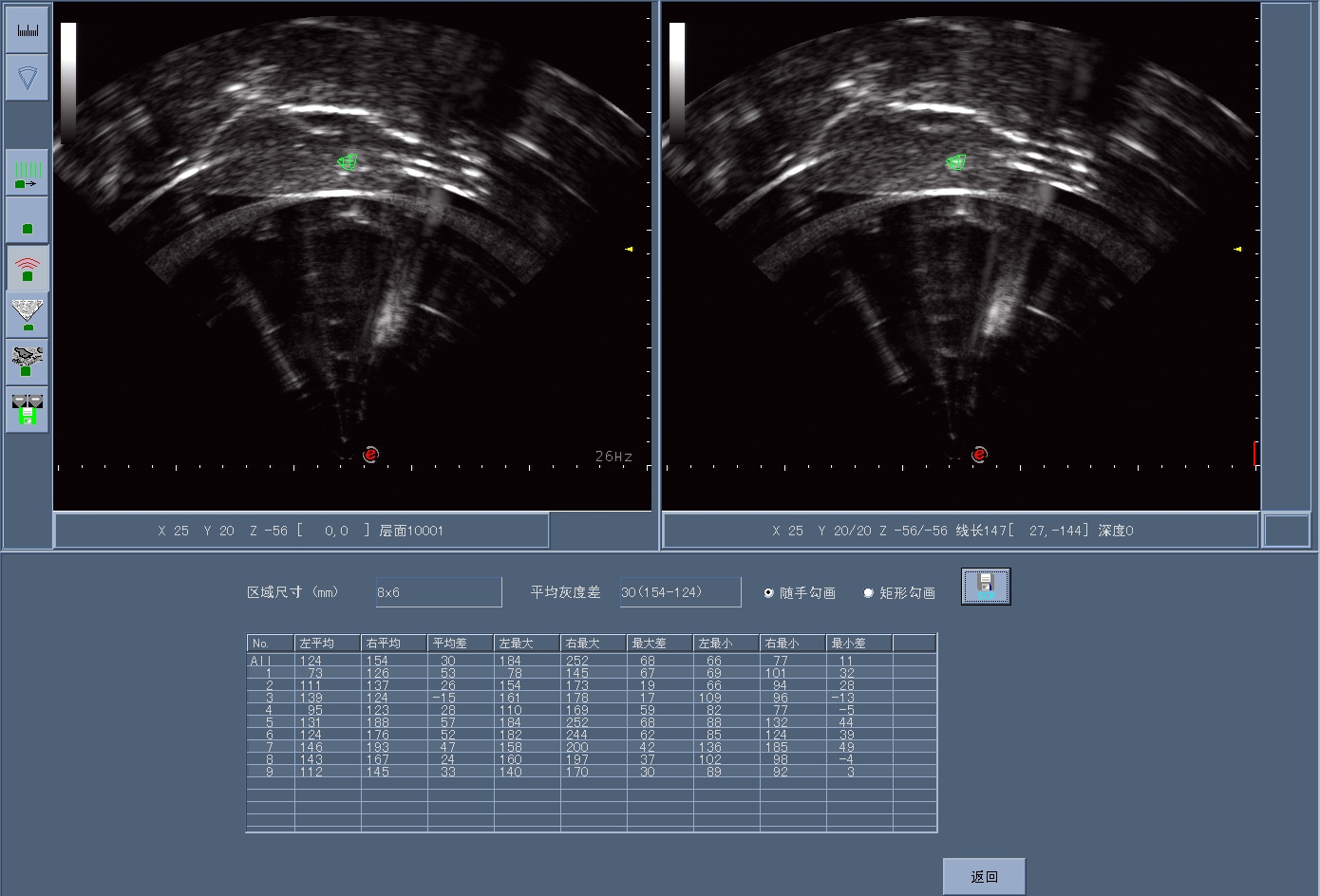

Supplement: Data S2 [file peerj-04-1716-s002.zip › raw data of excised bovine liver/PBS,150w,5s.jpg]

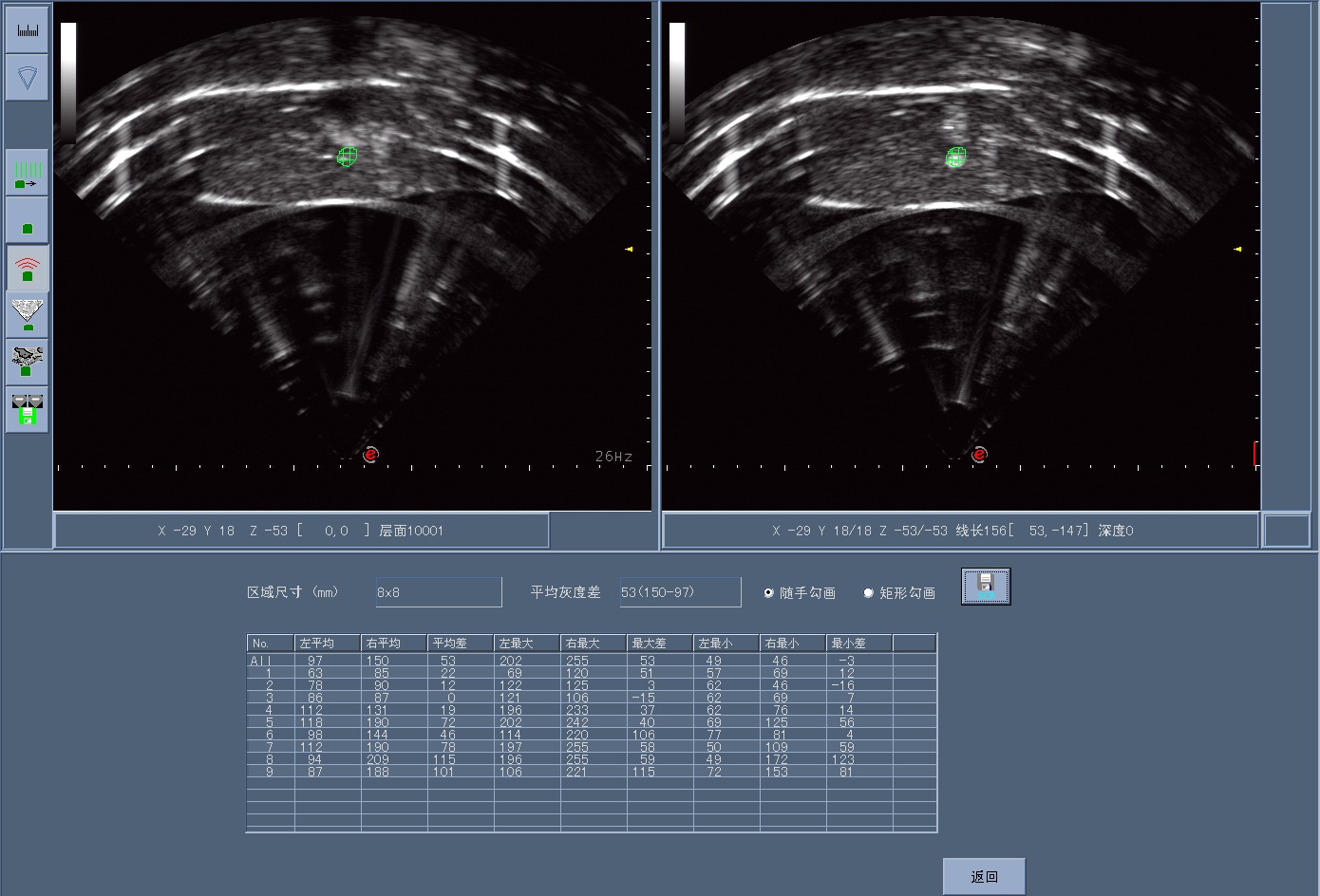

Supplement: Data S2 [file peerj-04-1716-s002.zip › raw data of excised bovine liver/PBS,180w,5s.jpg]

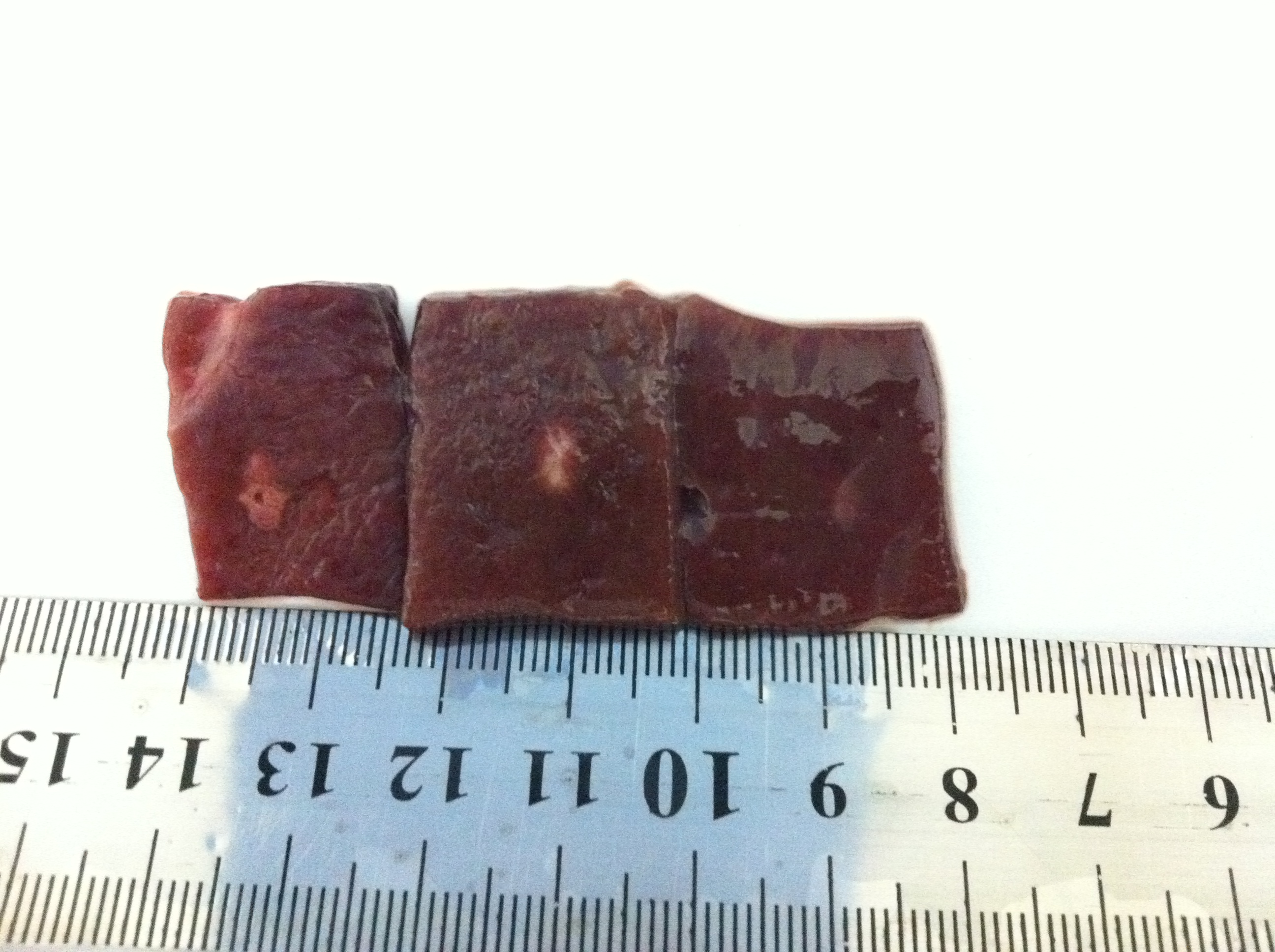

Supplement: Data S2 [file peerj-04-1716-s002.zip › raw data of excised bovine liver/pbs.JPG]

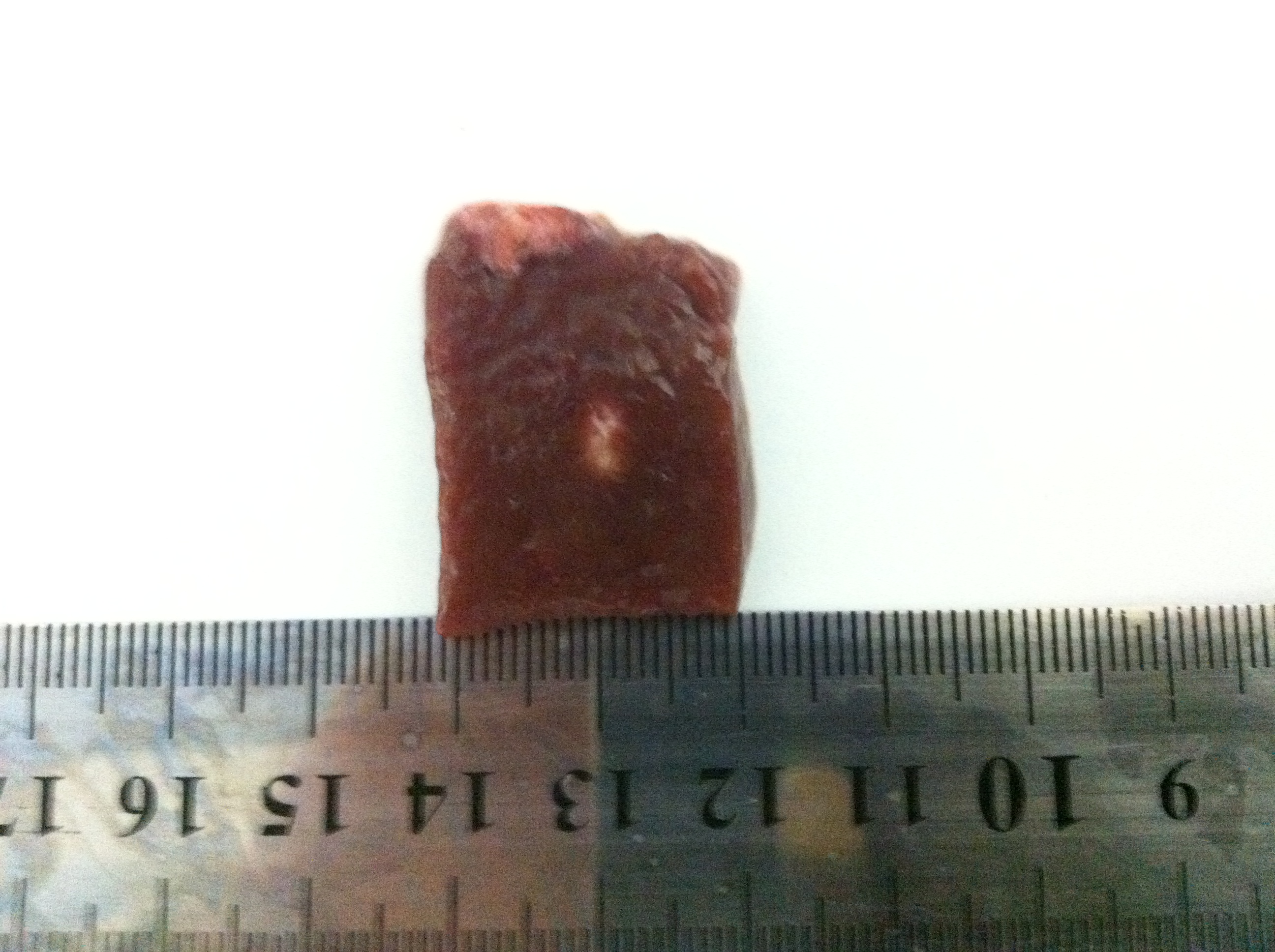

Supplement: Data S2 [file peerj-04-1716-s002.zip › raw data of excised bovine liver/pbs1.JPG]

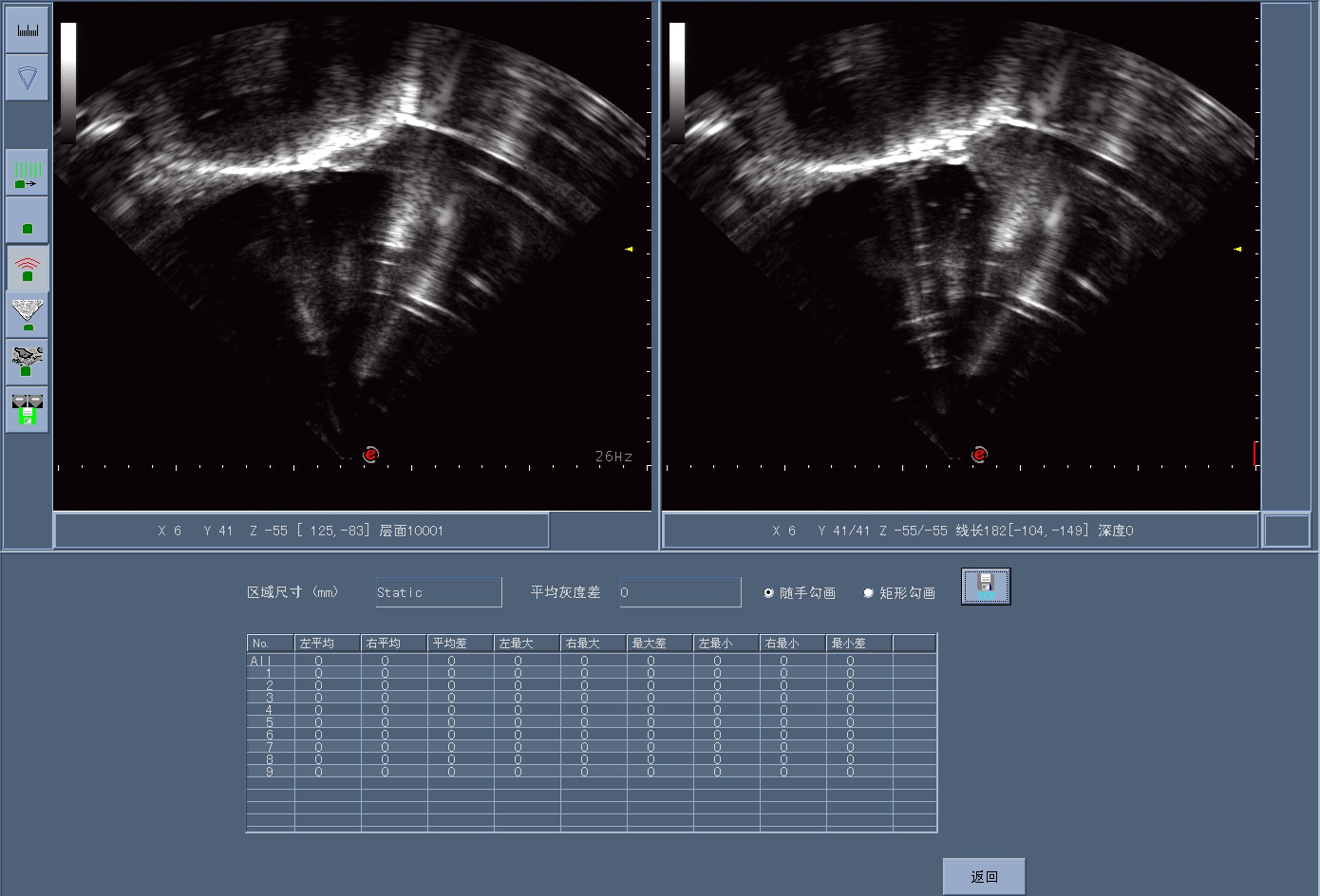

Supplement: Data S3 [file peerj-04-1716-s003.zip › raw data of rabbit breast tumor/11.jpg]

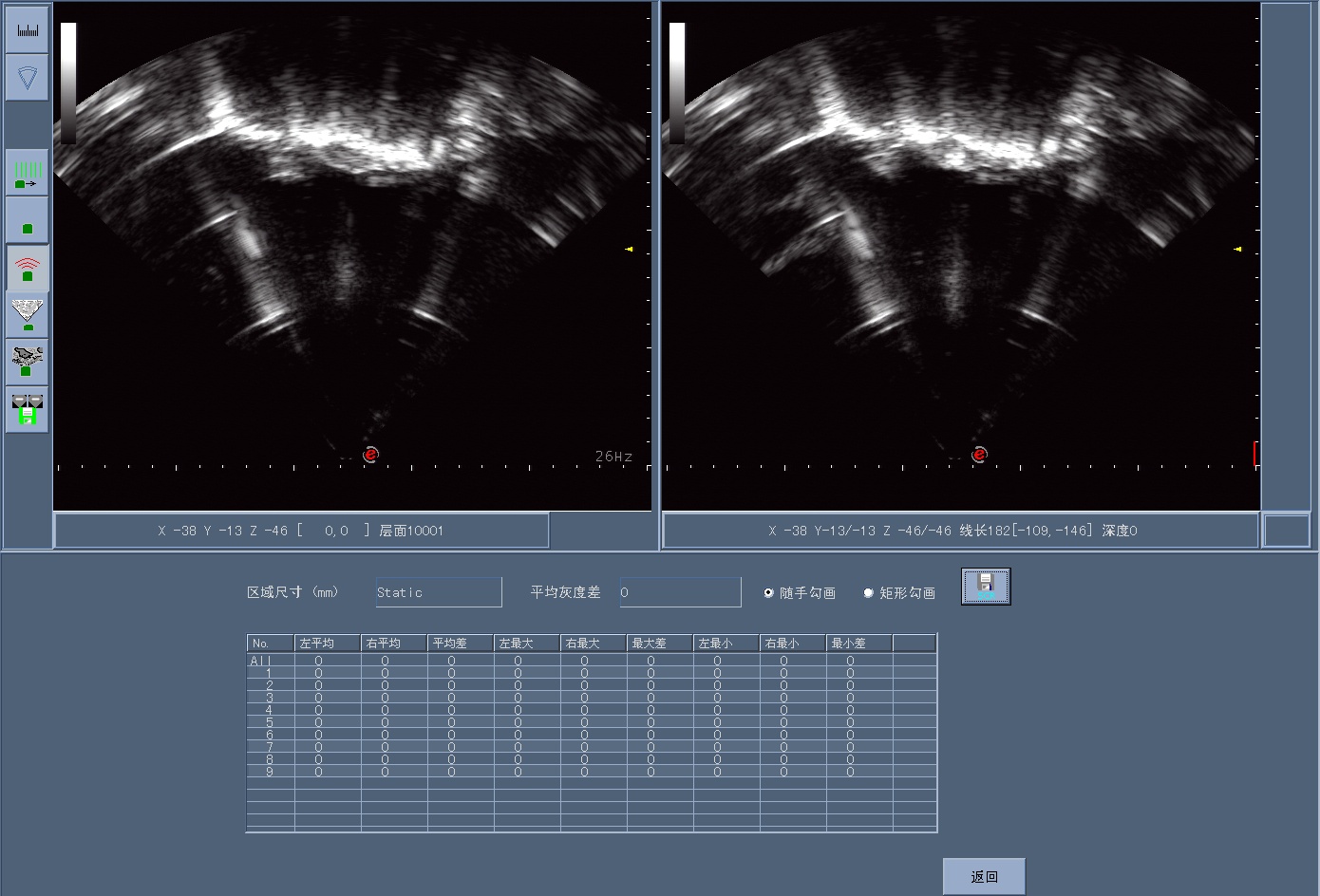

Supplement: Data S3 [file peerj-04-1716-s003.zip › raw data of rabbit breast tumor/13.jpg]

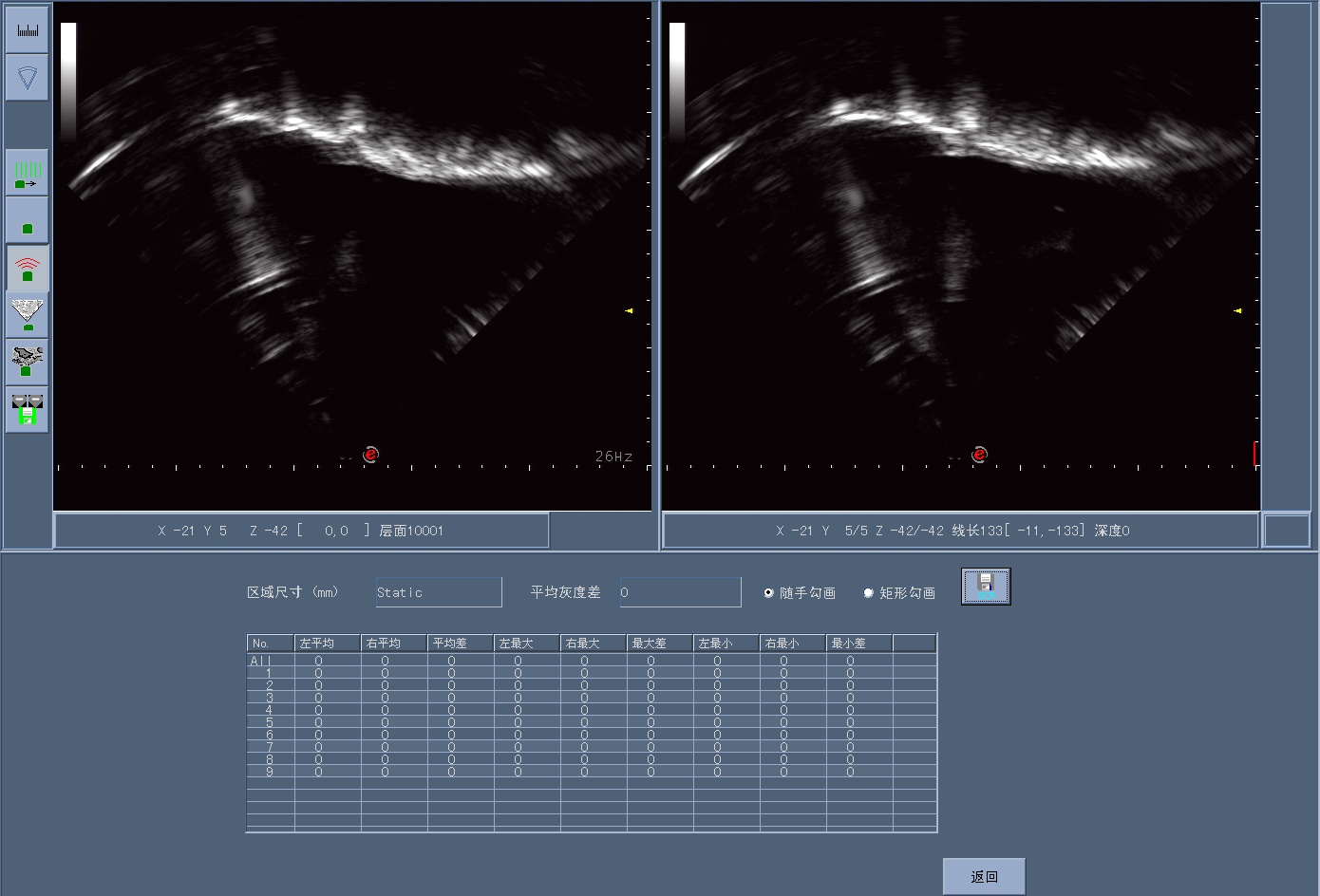

Supplement: Data S3 [file peerj-04-1716-s003.zip › raw data of rabbit breast tumor/7.jpg]

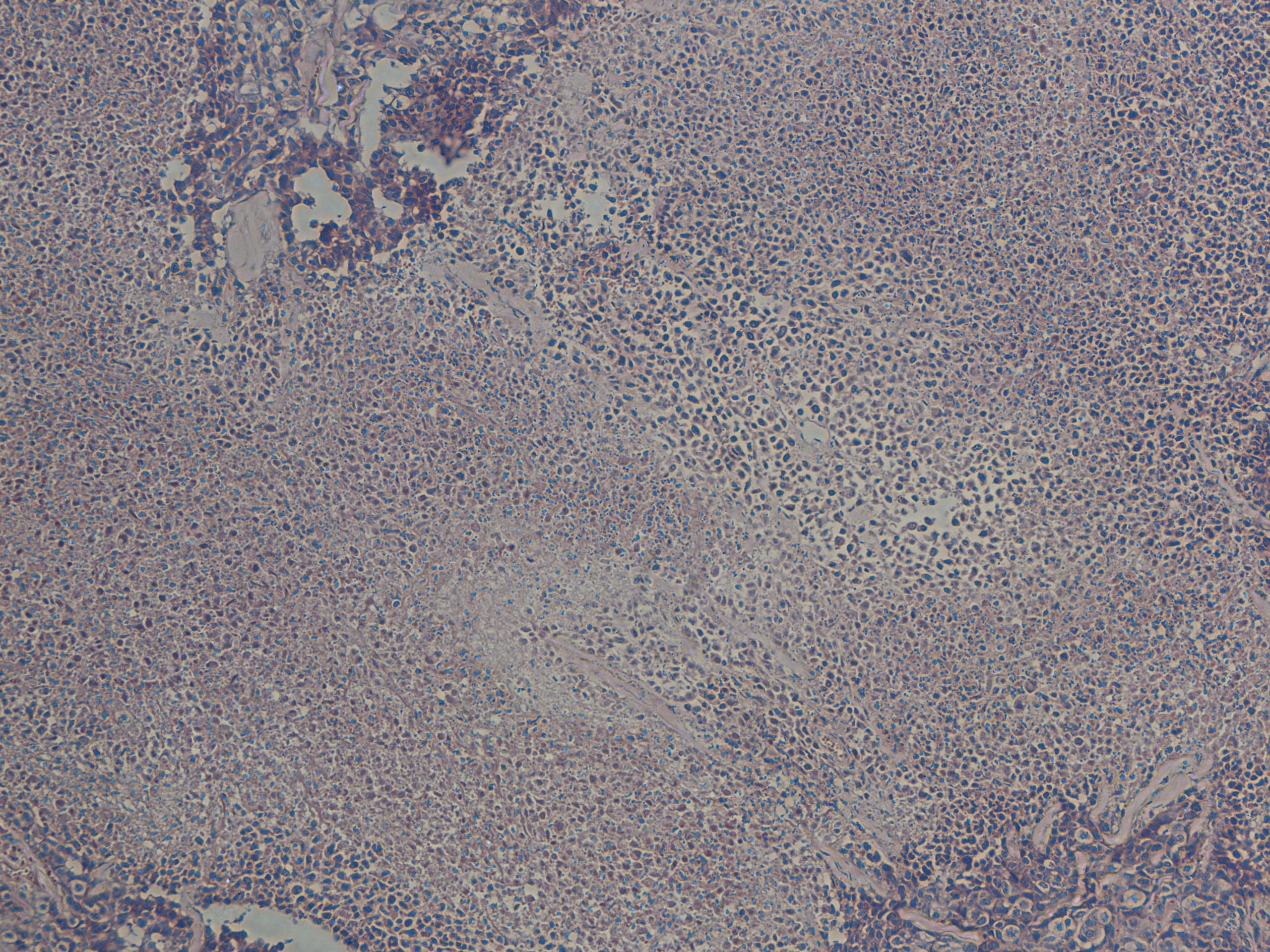

Supplement: Data S3 [file peerj-04-1716-s003.zip › raw data of rabbit breast tumor/Captured-100-1 (2).jpg]

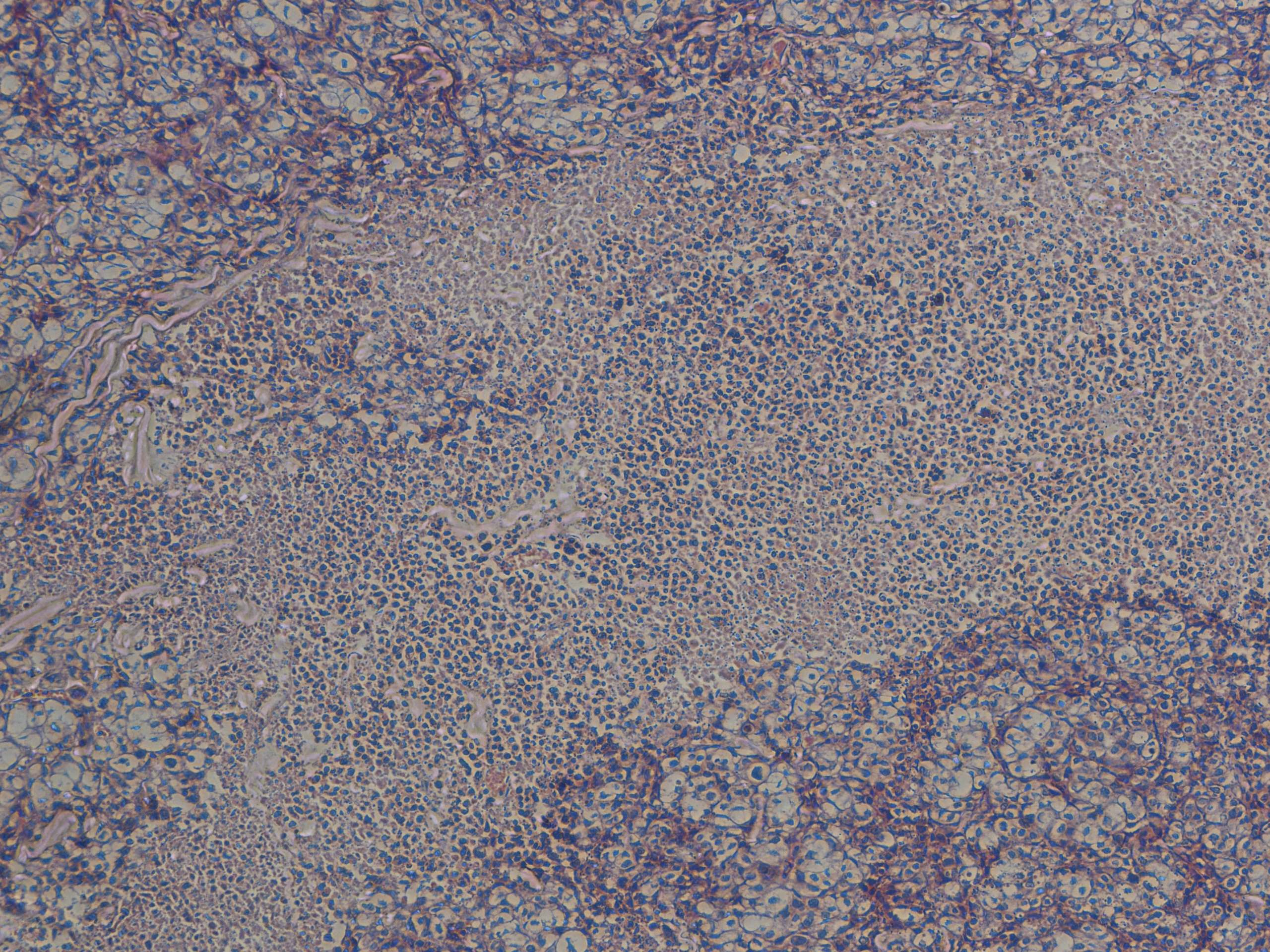

Supplement: Data S3 [file peerj-04-1716-s003.zip › raw data of rabbit breast tumor/Captured-100-2 .jpg]

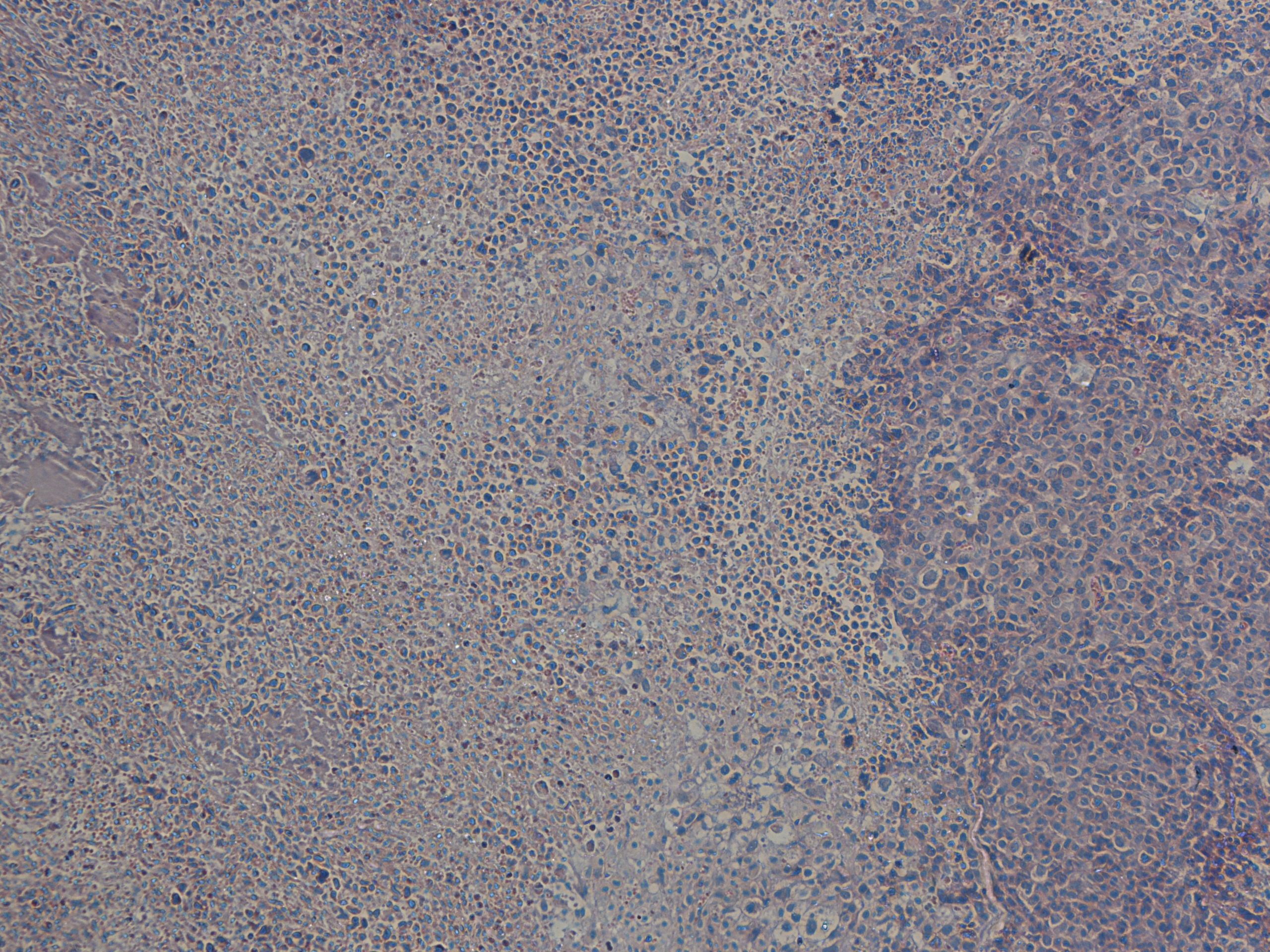

Supplement: Data S3 [file peerj-04-1716-s003.zip › raw data of rabbit breast tumor/Captured-100.jpg]

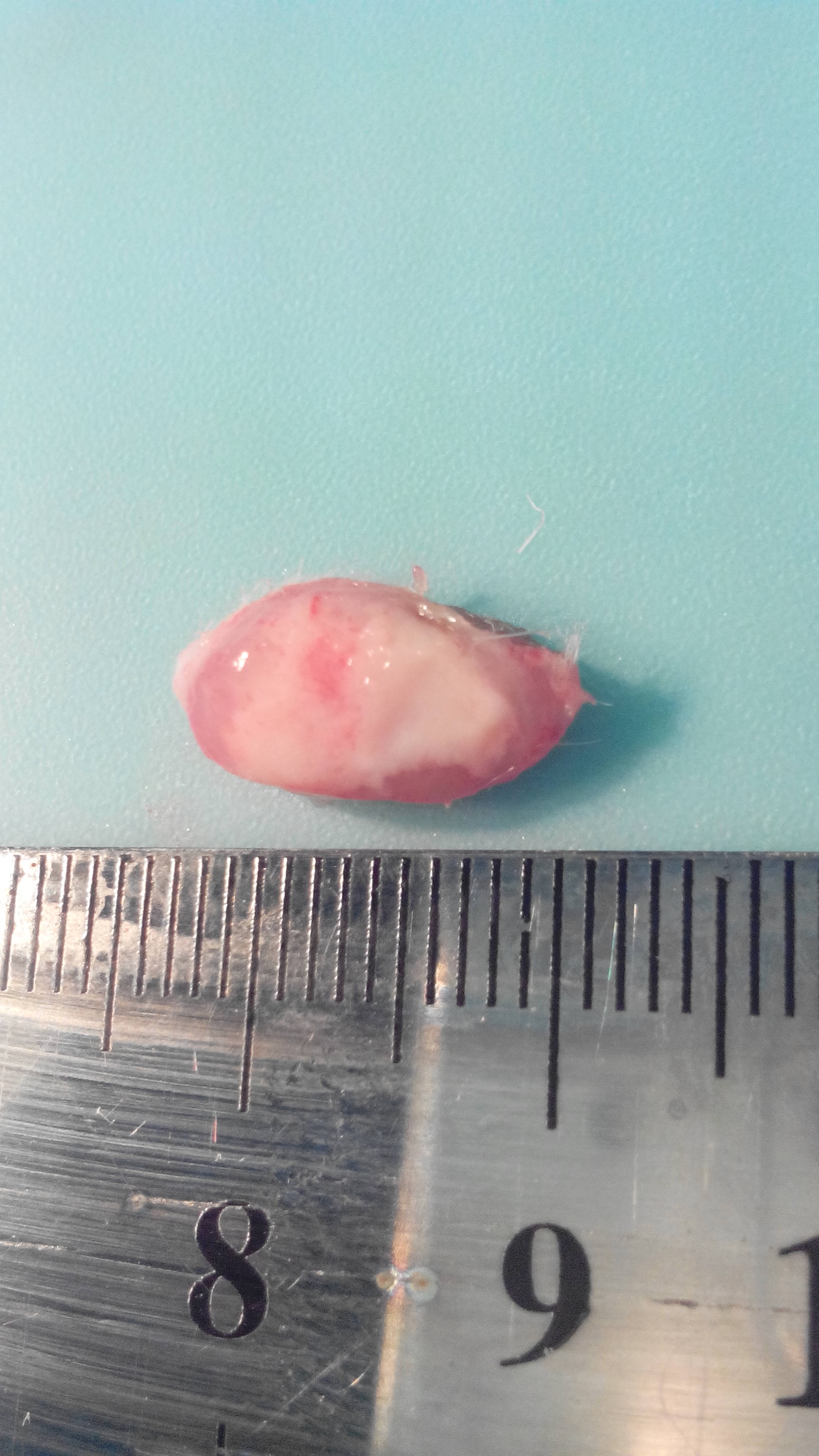

Supplement: Data S3 [file peerj-04-1716-s003.zip › raw data of rabbit breast tumor/IMG_20141105_192625.jpg]

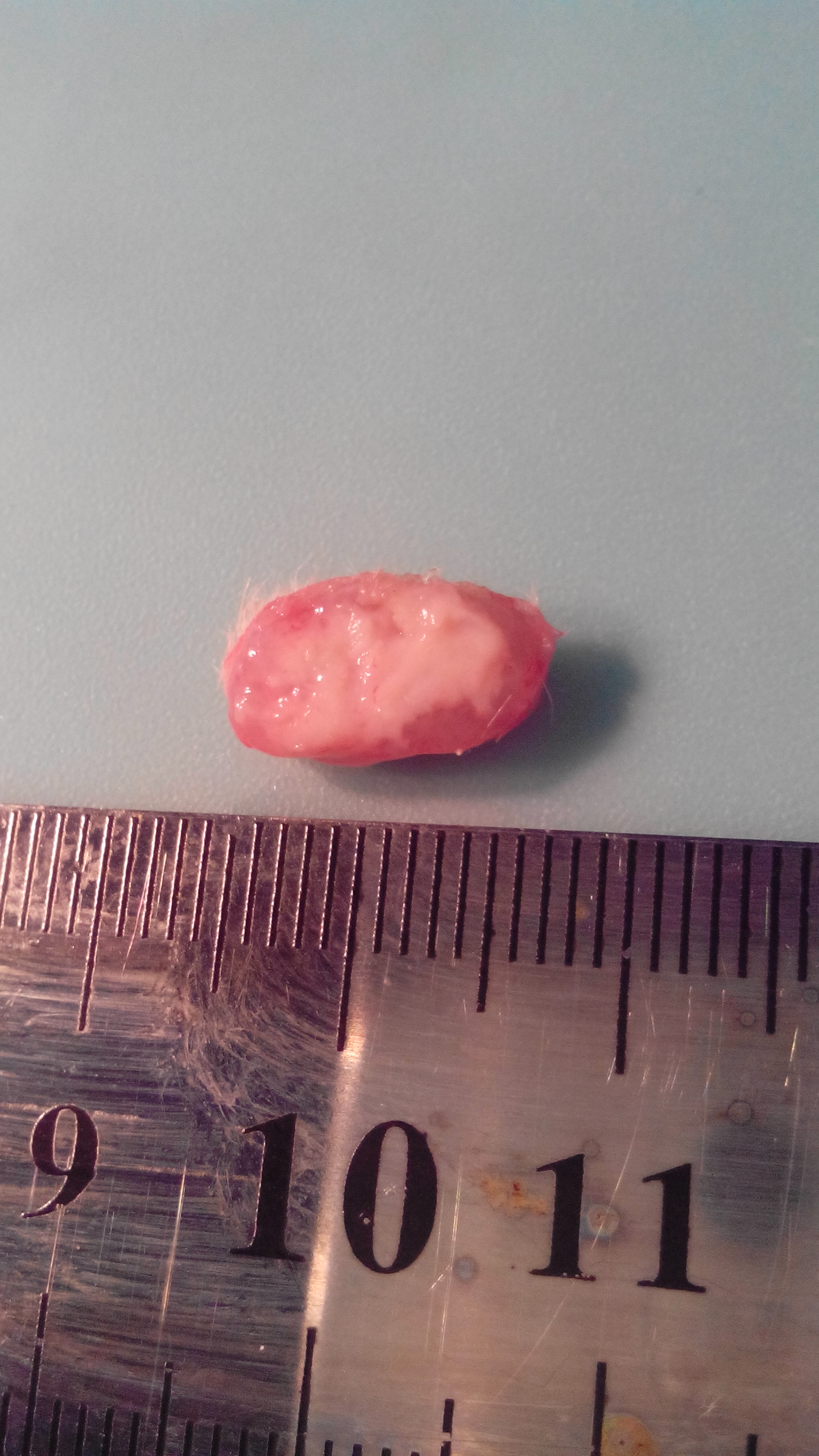

Supplement: Data S3 [file peerj-04-1716-s003.zip › raw data of rabbit breast tumor/IMG_20141105_193132.jpg]

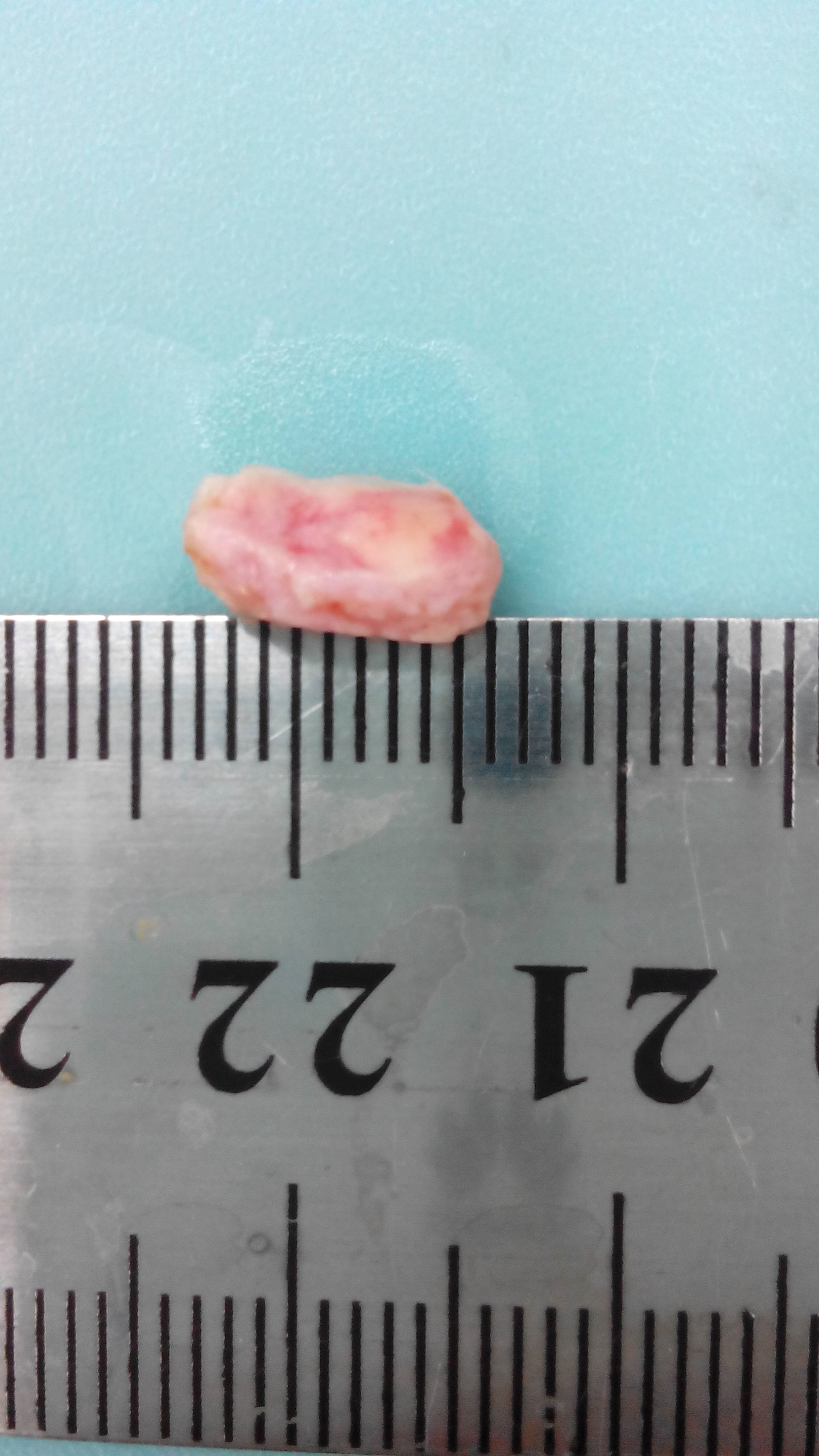

Supplement: Data S3 [file peerj-04-1716-s003.zip › raw data of rabbit breast tumor/IMG_20141106_150925.jpg]

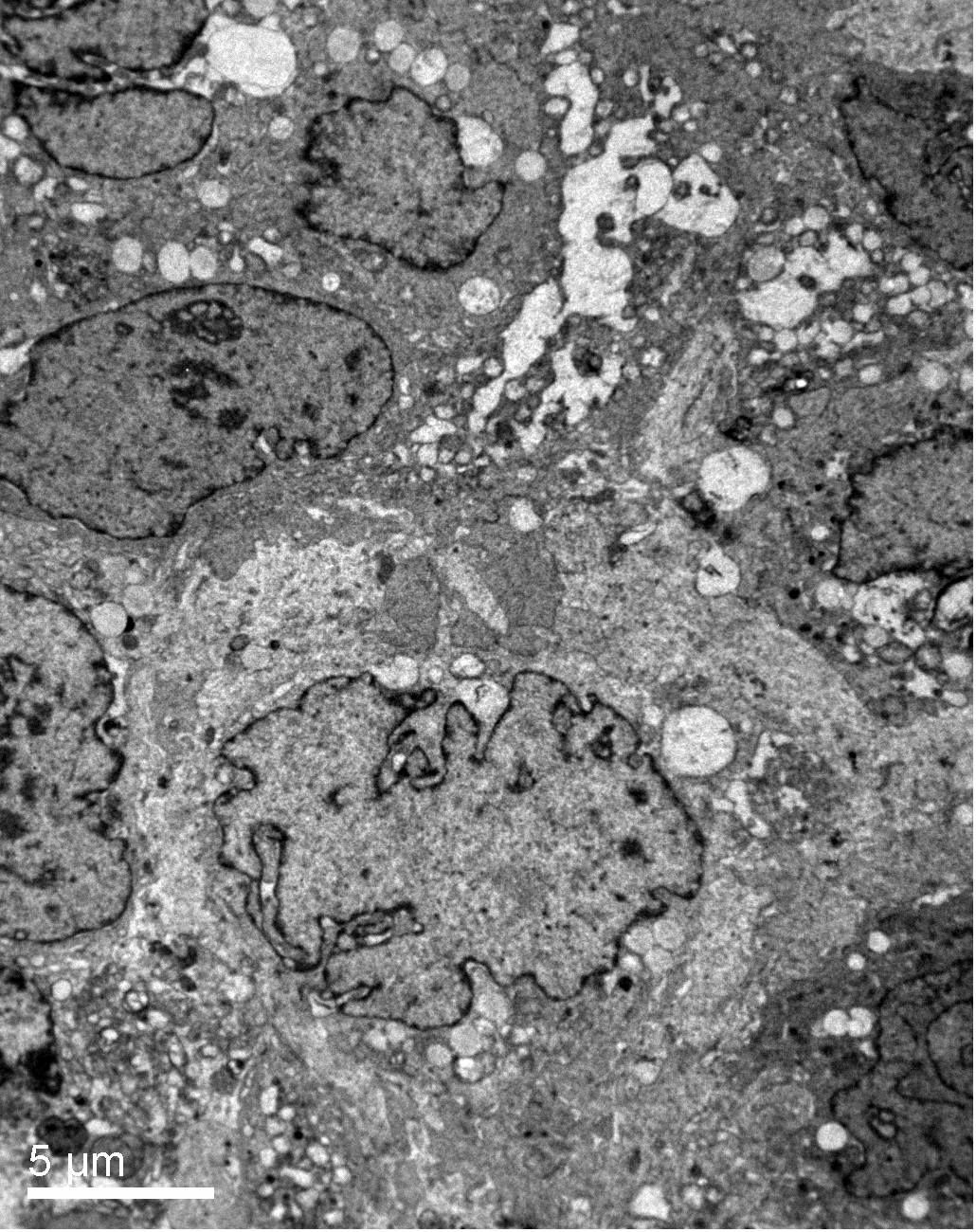

Supplement: Data S3 [file peerj-04-1716-s003.zip › raw data of rabbit breast tumor/j14005-1-13-3500.png]

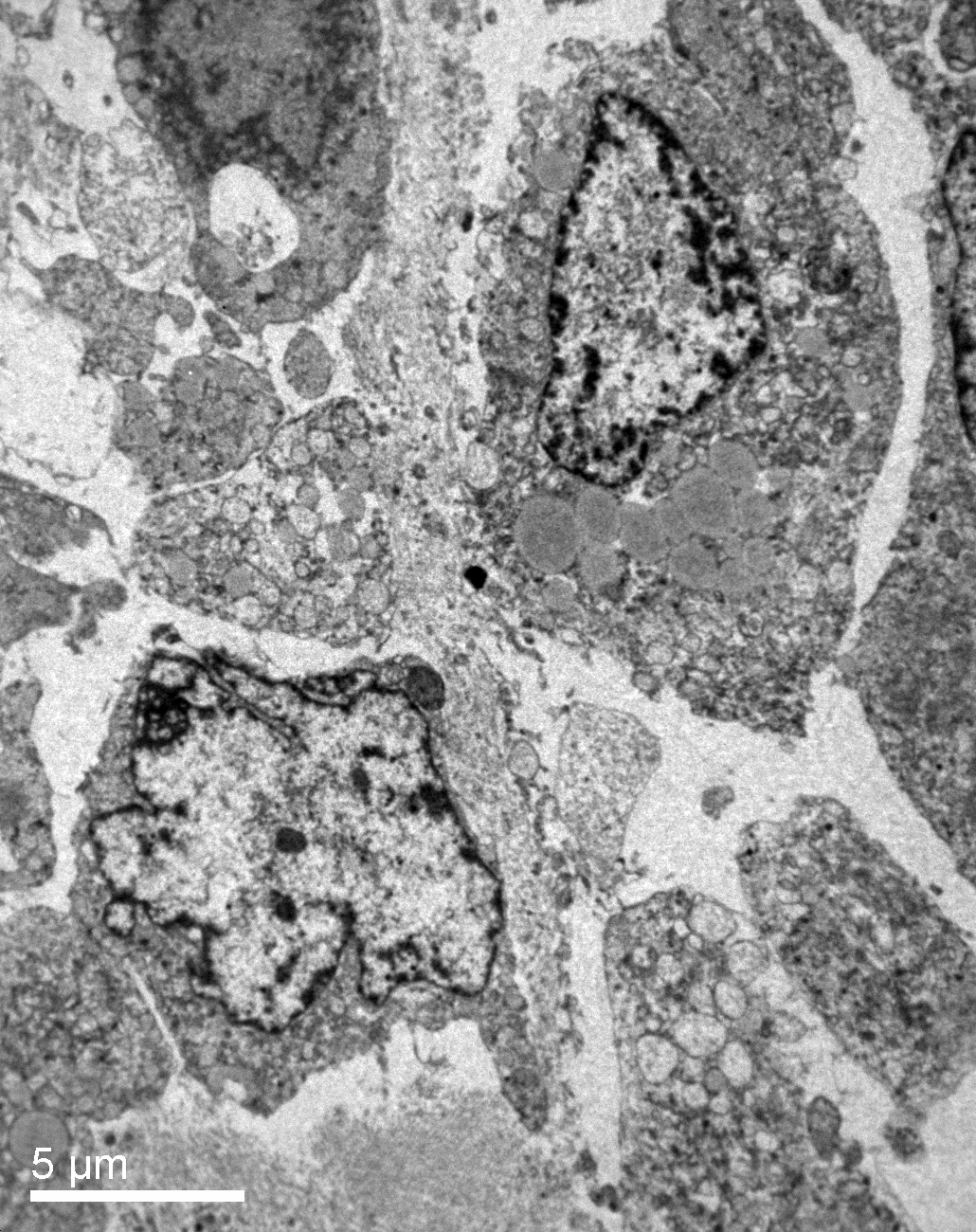

Supplement: Data S3 [file peerj-04-1716-s003.zip › raw data of rabbit breast tumor/j14007-2-1-4000.png]

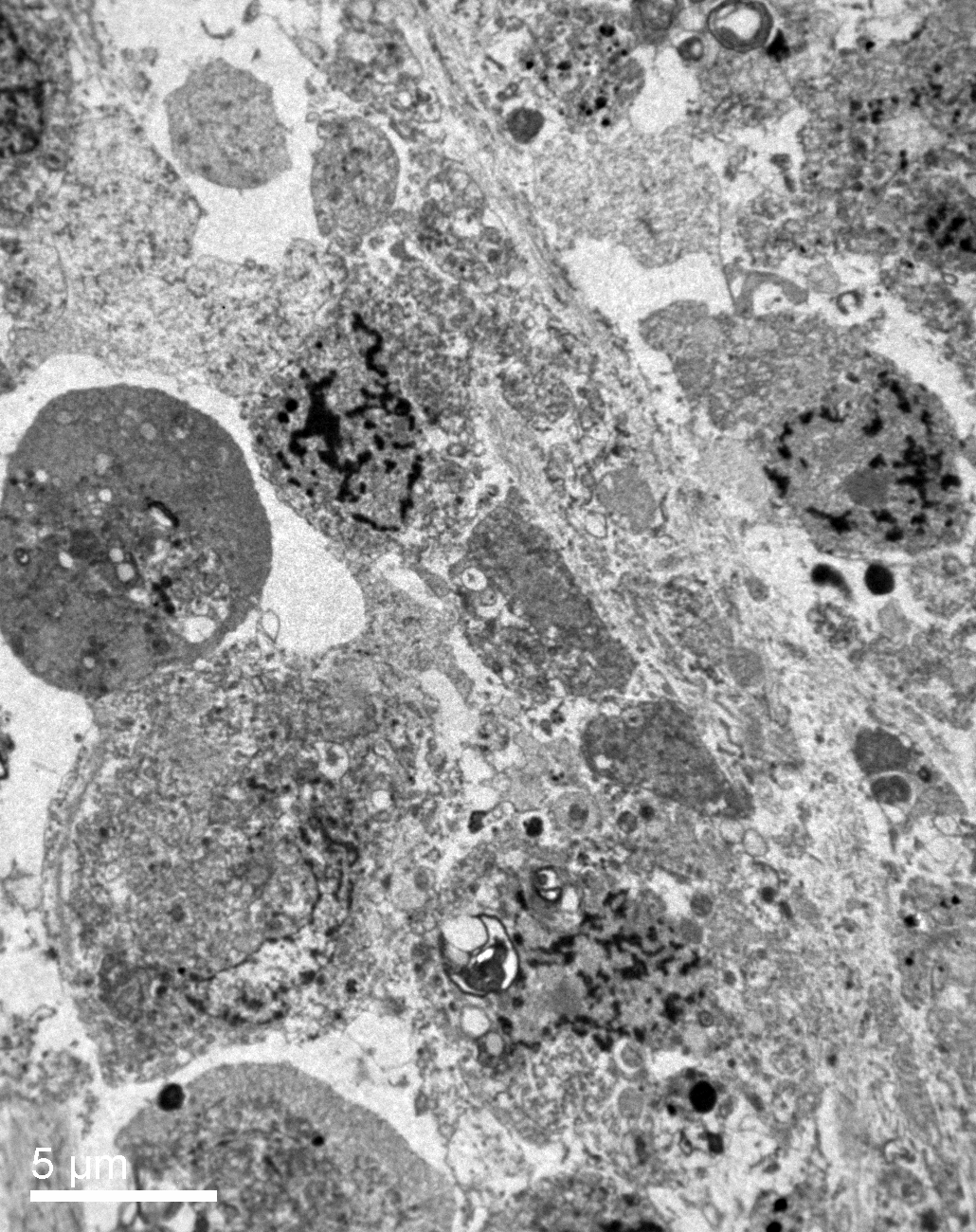

Supplement: Data S3 [file peerj-04-1716-s003.zip › raw data of rabbit breast tumor/j14007-2-5-3500.png]

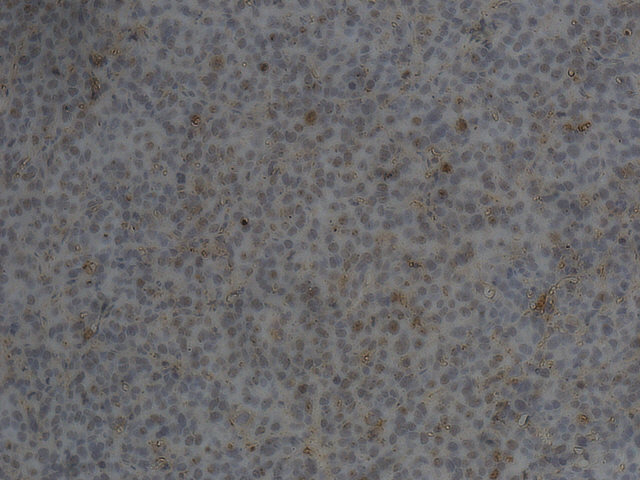

Supplement: Data S3 [file peerj-04-1716-s003.zip › raw data of rabbit breast tumor/┼─╔π-200-2.jpg]

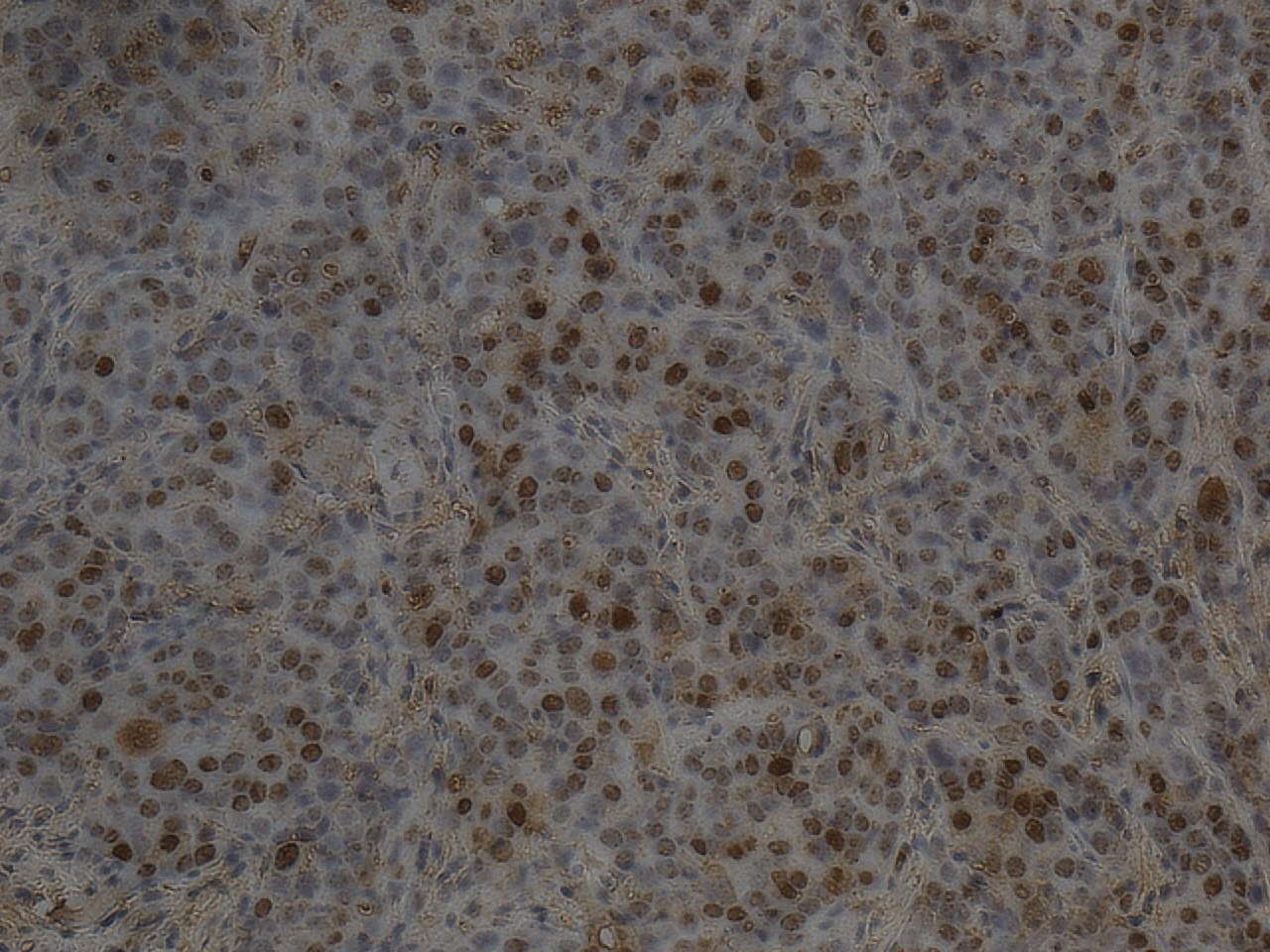

Supplement: Data S3 [file peerj-04-1716-s003.zip › raw data of rabbit breast tumor/┼─╔π-200.jpg]

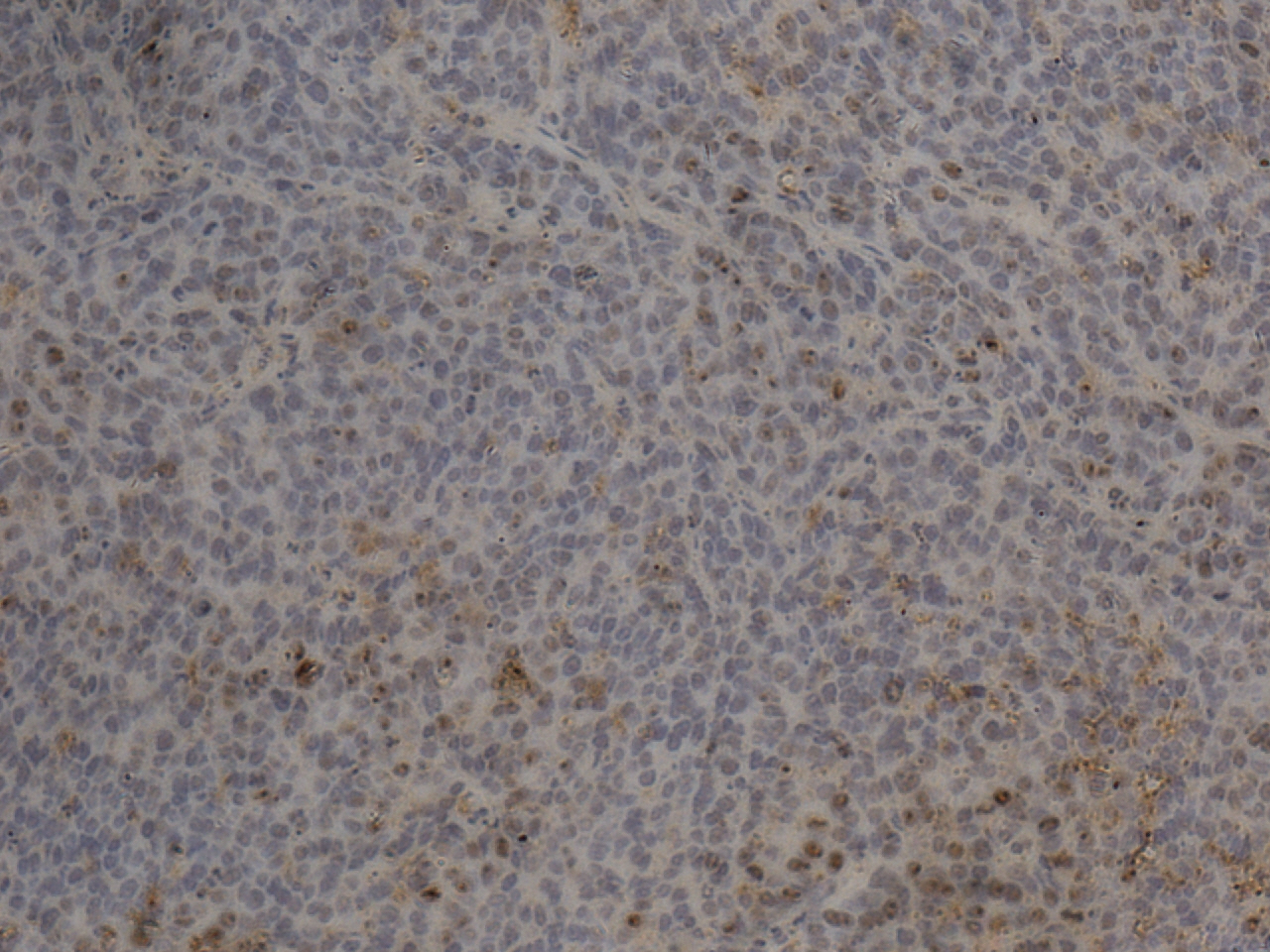

Supplement: Data S3 [file peerj-04-1716-s003.zip › raw data of rabbit breast tumor/┼─╔π-20011.jpg]
